# Environment-dependent mutualism–parasitism transitions in the incipient symbiosis between *Tetrahymena utriculariae* and *Micractinium tetrahymenae*

**DOI:** 10.1093/ismejo/wraf203

**Published:** 2025-09-06

**Authors:** Md Mostafa Kamal, Yu-Hsuan Cheng, Li-Wen Chu, Phuong-Thao Nguyen, Chien-Fu Jeff Liu, Chia-Wei Liao, Thomas Posch, Jun-Yi Leu

**Affiliations:** Institute of Molecular Biology, Academia Sinica, Taipei 115, Taiwan; Institute of Molecular Biology, Academia Sinica, Taipei 115, Taiwan; Morgridge Institute for Research, University of Wisconsin-Madison, Madison, WI 53715, United States; Institute of Molecular Biology, Academia Sinica, Taipei 115, Taiwan; Institute of Molecular Biology, Academia Sinica, Taipei 115, Taiwan; Department of Life Sciences, National Central University, Taoyuan 320, Taiwan; Institute of Molecular Biology, Academia Sinica, Taipei 115, Taiwan; Institute of Molecular Biology, Academia Sinica, Taipei 115, Taiwan; Limnological Station, Department of Plant and Microbial Biology, University of Zurich, Kilchberg, Zurich 8802, Switzerland; Institute of Molecular Biology, Academia Sinica, Taipei 115, Taiwan

**Keywords:** ciliate, endosymbiosis, genome assembly, mutualism, parasitism

## Abstract

Mutualistic endosymbiosis is a cornerstone of evolutionary innovation, enabling organisms to exploit diverse niches unavailable to individual species. However, our knowledge about the early evolutionary stage of this relationship remains limited. The association between the ciliate *Tetrahymena utriculariae* and its algal endosymbiont *Micractinium tetrahymenae* indicates an incipient stage of photoendosymbiosis. Although *T. utriculariae* cells rely on endosymbiotic algae to grow in low-oxygen conditions*,* they gradually lose the endosymbionts in oxic conditions. In this study, comparative phylogenomics revealed accelerated evolution in mitochondrial DNA and nucleus-encoded mitochondrial genes in *T. utriculariae*. Symbiotic cells displayed elongated mitochondria that interacted intimately with endosymbionts. Inhibition of mitochondrial fatty acid oxidation reduced host fitness but increased the endosymbiont population. Time-series transcriptomics revealed physiological fine-tuning of the host across day–night cycles, highlighting symbiosis-associated regulatory adjustments. Endosymbiotic algae downregulated photosynthesis-related genes compared with free-living cells, which correlated with reduced chlorophyll content, suggesting a shift toward host resource exploitation to compensate for diminished photosynthetic capacity. Under oxic conditions, symbiotic *T. utriculariae* cells exhibited lower fitness than aposymbiotic cells. Our results demonstrate that incipient endosymbioses employ mitochondrial remodeling and endosymbiont metabolic reprogramming to actively regulate transitions between mutualistic and parasitic states, revealing how symbiotic partnerships navigate environmental pressures during their incipient stage of evolutionary establishment.

## Introduction

Endosymbiosis, the intimate association by which one organism lives within the cells of another, has been a driving force in the evolution of complex life on Earth. The most profound examples are the origins of mitochondria and chloroplasts from free-living bacteria, events that gave rise to eukaryotic cells and enabled the diversification of plants and animals [1, 2]. These organelles are indispensable for energy production and photosynthesis, highlighting the evolutionary significance of endosymbiotic relationships.

Photoendosymbiosis, a specific type of endosymbiosis involving a photosynthetic organism residing within a nonphotosynthetic host, is widespread across various taxa. This relationship is fundamental in marine ecosystems, particularly in coral reefs, where cnidarians harbor photosynthetic dinoflagellates of the genus *Symbiodinium*. The endosymbionts provide the host with organic compounds produced via photosynthesis, whereas the host offers protection and access to light and inorganic nutrients [3, 4]. Similar mutualistic associations are present in freshwater environments, such as the endosymbiosis between the ciliate *Paramecium bursaria* and green algae of the genus *Chlorella*. In this case, the algae contribute photosynthetically derived oxygen and nutrients, such as maltose, and, in return, they receive shelter and carbon dioxide from the host [5, 6]. Beyond ciliates, photosymbiotic relationships span diverse protist lineages, including amoebozoans, where fitness benefits are condition-dependent [7].

Stable photoendosymbiotic relationships are characterized by a high degree of metabolic integration and mutual dependence, arising from long-term coevolution [8, 9]. The genomes of the host and endosymbiont often exhibit signs of reciprocal gene transfer and functional specialization, facilitating efficient nutrient exchange and adaptation to specific ecological niches [10, 11]. When an endosymbiotic relationship is disrupted, the fitness of both the host and endosymbiont is often compromised. However, the initial stages of endosymbiosis, when the interactions between host and endosymbiont are less integrated, remain poorly understood. Investigating nascent or early evolutionary-stage symbiotic relationships is crucial to elucidate the mechanisms and selective pressures that drive the evolution of stable mutualisms [12, 13].

The ciliate *Tetrahymena utriculariae* and its algal endosymbiont *Micractinium tetrahymenae* represent a unique system for studying the early evolutionary stage of photoendosymbiosis. *T. utriculariae* is a freshwater protozoan found initially in the traps of a carnivorous plant, *Utricularia reflexa*, where it plays a role in nutrient cycling [14]. *Micractinium tetrahymenae*, a green alga, has been identified as an endosymbiont within *T. utriculariae* [15]. The endosymbiotic algae can be transmitted vertically to progeny during cell division. However, when *T. utriculariae* cells are grown under oxic conditions and with bacterial food in saturation, they gradually lose their endosymbionts [15]. This scenario contrasts with that of the *P. bursaria* system, in which endosymbionts are eliminated only when chemicals or a lack of light compromise algal growth [16–18]. Thus, the endosymbiotic relationship between *T. utriculariae* and *M. tetrahymenae* appears relatively unstable, likely representing an incipient stage in its evolution.

Environmental factors play a crucial role during the evolution of endosymbiosis. Many endosymbionts provide their hosts with unique metabolites, enabling them to exploit specialized ecological niches. For instance, aphids rely on the obligate endosymbiont *Buchnera aphidicola* to synthesize essential amino acids that are absent from their phloem sap diet, thereby allowing them to flourish in nutrient-poor environments [19]. Similarly, deep-sea clams harbor symbionts with reduced genomes that oxidize hydrogen sulfide to provide chemical energy, enabling their survival in extreme and resource-limited habitats [20]. These marine and insect examples underscore how a stable and constant selective environment can promote tight host-symbiont coevolution, leading to enduring and mutually beneficial relationships, a concept comprehensively reviewed previously [21], which elaborates on the genomic intricacies underpinning these associations. By contrast, shifts in environmental conditions can destabilize these interactions, even leading to the complete breakdown of symbiosis, as detailed in previous work on evolutionary transitions in bacterial symbioses [22]. Such condition-dependent relationships likely occur during the early stages of symbiotic integration when the partners are not yet fully co-adapted.

*Tetrahymena* species have been studied for over a century, with *T. thermophila* serving as a model organism in molecular and cellular biology [23, 24]. Currently, *T. utriculariae* is the only *Tetrahymena* species known to form endosymbiotic relationships with green algae naturally. However, experimental evolution studies have shown that under laboratory conditions, *Tetrahymena* species such as *T. thermophila* can also establish transient endosymbiotic associations with algae. In these instances, the algae have evolved to produce more carbohydrates, which benefits the host [25, 26]. The wealth of knowledge on the cell biology of *Tetrahymena* species provides an excellent resource to dissect these endosymbiotic relationships in detail.

In this study, we characterized the genomic and transcriptomic profiles of both *T. utriculariae* and *M. tetrahymenae* to identify possible pathways and physiological changes involved in their symbiotic interaction. Our phylogenetic analysis showed that mitochondria-related genes in *T. utriculariae* have undergone accelerated evolution compared to those in closely related *Tetrahymena* species. Moreover, our analyses of mitochondrial morphology and gene expression revealed specific changes in symbiotic *T. utriculariae* cells, indicating that the host mitochondria have evolved to adapt to the endosymbionts. Many genes involved in photosynthesis were downregulated in the endosymbiotic algae, and their chlorophyll content was also reduced. This scenario raised the possibility that *M. tetrahymenae* sequesters host nutrients to support its own growth. Consistently, we observed that symbiotic *T. utriculariae* cells grew more slowly than aposymbiotic cells when cultured under oxic conditions with bacterial food in saturation. Our data indicate that a symbiotic relationship can switch from being mutualistic to parasitic depending on the environment, providing insights into the early stages of endosymbiotic evolution.

## Materials and methods

### Strains and culture conditions

*Tetrahymena utriculariae* was maintained in lettuce medium with *Klebsiella pneumoniae* (NBRC 100048) under a 12 h-light/12-h dark cycle. Growth assays compared symbiotic and aposymbiotic cells under oxic and hypoxic conditions; hypoxia was generated by culturing in airtight, filled vials. Free-living *M. tetrahymenae* was isolated from the symbiosis by plating on CA (a freshwater algal culture medium developed by the National Institute for Environmental Studies, Japan) agar, restreaked on CA + ampicillin (100 μg/ml), and propagated in CA + ampicillin (100 μg/ml) liquid culture.

### Genome sequencing and annotation

The genomes of *T. utriculariae* and *M. tetrahymenae* were sequenced using long-read platforms, including PacBio and Oxford Nanopore, in conjunction with Illumina short reads. Assemblies were generated with Canu and polished with Pilon and NextPolish. Gene prediction was performed using BRAKER2 with RNA-seq and protein hints; completeness was assessed using BUSCO (assembly metrics were obtained via QUAST).

### RNA sequencing and analysis

Total RNA (*n* = 3 biological replicates per condition) was extracted with TRI reagent and column cleanup, strand-specific libraries were prepared with the Agilent SureSelect kit, and 150 bp paired-end reads were generated on a NovaSeq 6000 System (Illumina). Reads were quality-checked and trimmed (FastQC/fastp), quantified (kallisto), and analyzed for differential expression with limma (|log₂FC| ≥ 1; adjusted *P* ≤ .01).

### Comparative genomics

Orthologous groups were identified with OrthoFinder; synteny was evaluated with MCScanX (via DupGen_finder); phylogenetic trees were inferred with RAxML-NG; functional enrichment was performed using Gene Ontology (GO) analyses.

### Cell biology assays

Cellular ultrastructure was examined by transmission electron microscopy; host control of endosymbiont load was tested with etomoxir; chlorophyll was quantified spectrophotometrically at 652 and 665 nm.

Complete protocols, software versions, and parameters are provided in the Supplementary text.

## Results

### High-quality macronuclear genome assembly and annotation of *T. utriculariae*

A chromosome-level macronuclear (MAC) genome of *T. utriculariae* was generated to understand the genetic basis of photoendosymbiosis. We assembled the genome from long- and short-read data, yielding 181 MAC chromosomes and a total size of 98.0 Mb (Table 1). Annotation identified 23 219 genes, with a mean coding sequence length of 2081 bp. BUSCO (Alveolata) recovered 97.7% of conserved genes, comparable to *T. thermophila* (97.1%; Table 1). These metrics indicated that the assembly captured the essential gene set for functional and comparative analyses.

**Table 1:** Comparative genomic attributes of *T. utriculariae*, *T. thermophila*, and *T. malaccensis*.

Species	Total assembled MAC chromosomes/contigs	Genome size (Mb)	N50 (Kb)	Shortest chromosome/contig (Kb)	Longest chromosome/contig (Mb)	Genomic GC content (%)	Gene number	Average length of protein-coding sequence (bp)	BUSCO completeness (%)
*T. utriculariae* (this study)	181	98.0	877	19.5	4.2	22.8	23 219	2081	97.7
*T. thermophila* [29]	181	103.3	930	21.2	3.3	22.3	26 258	1844	97.1
*T. malaccensis* [30]	554 (contig level only)	106.6	496	1.0	2.2	22.0	26 378	1744	99.4

**Figure 1:**
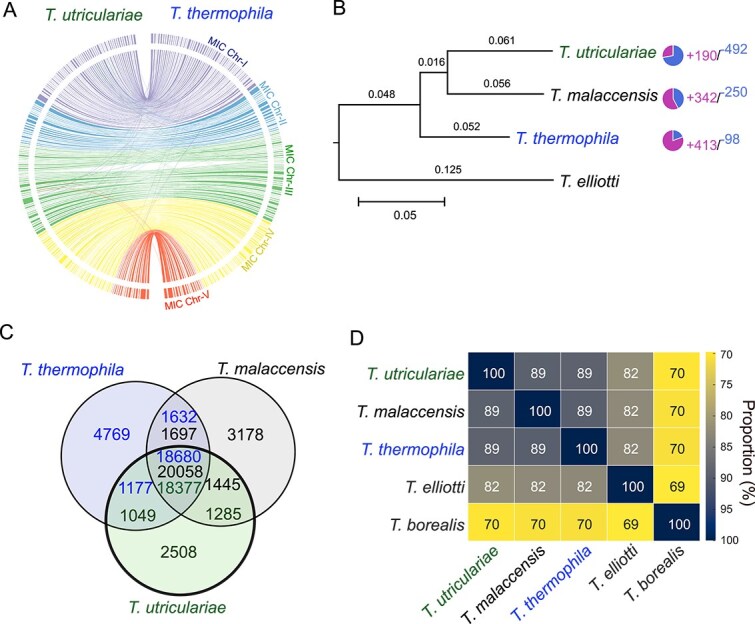
Comparative genomic analysis of *T. utriculariae* in relation to closely related ciliate species. (A) Circos plot of synteny between *T. utriculariae* (left) and *T. thermophila* (right). Outer rings show macronuclear (MAC) chromosomes, color-coded by originating micronuclear (MIC) chromosome (MIC Chr I-V). Inner links denote syntenic blocks (>2000 bp). (B) Maximum-likelihood phylogeny from orthologous genes for four *Tetrahymena* species; branch lengths indicate genetic distance (scale bar: 0.05 substitutions per site). Pie charts summarize gene-family dynamics: segments represent expansions (+190, +342, and +413) and contractions (−492, −250, and −98) for *T. utriculariae*, *T. malaccensis*, and *T. thermophila*, respectively. *T. elliotti* is the outgroup. (C) Venn diagram of shared and unique orthologous gene clusters among *T. utriculariae* (green), *T. malaccensis* (black), and *T. thermophila* (blue); numbers indicate counts in each set. (D) Average amino acid identity (AAI) heatmap across five *Tetrahymena* species; cells show pairwise percentage identity.

Synteny analysis uncovered high collinearity (conserved gene order) between *T. utriculariae* and *T. thermophila* (Fig. 1A), indicating that most genomic regions have remained relatively stable throughout their evolutionary history. However, the synteny plot also revealed genomic rearrangements. These regions of divergence may reflect species-specific adaptations. Gene-level examples of conserved synteny and species-specific rearrangements are presented in the Comparative genomics section below.

To clarify the evolutionary relationships between *T. utriculariae* and other species within the *Tetrahymena* genus, we used our genomics data to conduct a phylogenetic analysis on 12 *Tetrahymena* species and an outgroup, *Ichthyophthirius multifiliis*, a close relative of the genus *Tetrahymena* [27, 28] (Supplementary Fig. S1A). The resulting phylogenetic tree positioned *T. utriculariae* in a distinct clade with *T. malaccensis* and *T. thermophila*. Divergence time estimates (Supplementary Fig. S1B) indicated that *T. utriculariae* and *T. malaccensis* diverged from their common ancestor with *T. thermophila* ~23 million years ago (Ma), and from each other around 19 Ma. To focus on this closely related clade, we reconstructed a phylogenetic tree using only *T. utriculariae*, *T. malaccensis*, and *T. thermophila*, with *T. elliotti* as an outgroup (Fig. 1B), which resulted in a similar phylogenetic pattern. Despite their genetic similarity, only *T. utriculariae* has evolved into a photoendosymbiotic species, indicating that this is a species-specific adaptation.

### Comparative genomics and protein similarity between *T. utriculariae* and other *Tetrahymena* species

We analyzed the differences between the genomes of *T. utriculariae* and its closely related species by conducting an orthologous group analysis. *T. utriculariae* shared >79% of its genes (18 377 out of 23 219, Supplementary Data S1A) with both *T. malaccensis* and *T. thermophila* (Fig. 1C, Supplementary Data S1B). An amino acid identity (AAI) analysis of 9430 one-to-one orthologous genes further confirmed the close relationship among these species. *T. utriculariae* shared 89% AAI with both *T. malaccensis* and *T. thermophila*, but the AAI decreased to 82% and 70%, respectively, with the following most closely related species, *T. elliotti* and *T. borealis* (Fig. 1D).

*Tetrahymena utriculariae* possesses 2508 unique genes, representing ~11% of its genome (Supplementary Data S1C). These genes are absent in both *T. malaccensis* and *T. thermophila*. Analyzing these *T. utriculariae*-specific genes could shed light on unique adaptations in this species. A GO enrichment analysis of these *T. utriculariae*-specific genes showed enrichment for biotic interactions, cellular signaling, cellular catabolism, and carbohydrate metabolism (Supplementary Fig. S2, Supplementary Data S2A).

The biotic interactions and cell signaling GO categories encompass responses to other organisms and stimuli, signal transduction, and cell communication processes, which are critical for coordinating responses to internal and external stimuli [31]. Consequently, these unique genes may help *T. utriculariae* manage and respond to its symbiotic algae. The cellular catabolism and carbohydrate metabolism GO categories included autophagy, autophagosome organization, glycogen, and glucan biosynthesis, which may be involved in maintaining homeostasis and coordinating metabolic exchanges within the host–symbiont relationship [32]. Together, these *T. utriculariae*-specific genes may play a role in adaptations related to *T. utriculariae*’s specialized photoendosymbiotic relationship with *M. tetrahymenae*.

Among the 2508 *T. utriculariae*-specific genes, 716 are located in nonsyntenic regions compared to 1060 nonspecific genes (Supplementary Table S1), which revealed a significant association between gene specificity and the presence of syntenic blocks (χ^2^ = 1735.33, *P* < .001). Nonsyntenic blocks typically occur near telomeres or regions hosting chromosomal rearrangements (Supplementary Fig. S3). Additionally, telomeric regions are prone to rapid genomic adaptation and restructuring [33, 34]. Large-scale chromosomal rearrangements can also lead to evolutionary changes and the formation of new genes [35, 36].

Examining the gene family expansions and contractions provided further insights into the evolutionary pressures that have shaped the *T. utriculariae* genome. The species has undergone 190 gene family expansions and 492 contractions (Fig. 1B, pie charts, Supplementary Data S2B and C), supporting both the development of new functional capabilities and the streamlining of certain genomic aspects. The expanded gene families were enriched in processes related to biotic interactions, organelle organization, ion transport and homeostasis, nucleotide metabolism, protein modification, and stress responses (Supplementary Fig. S2, Supplementary Data S2D). These expansions plausibly reflect host adaptations to life inside *Utricularia* traps and to hosting photosynthetic endosymbionts. For example, the enrichment of diverse membrane transporters (e.g. nutrient exporters and importers) and ion homeostasis genes implies enhanced exchange and ionic regulation between host and symbiont or prey. This pattern is consistent with those of other protist–algal symbioses. For instance, *Mesodinium rubrum* and its cryptophyte endosymbiont co-express nutrient transporters, such as ammonium transporters, to shuttle nitrogen sources between partners​ [37]. These gene family expansions may facilitate nutrient exchange with the endosymbiont, helping the host cope with the stress of low oxygen levels. Similar genomic adaptations have been observed in other organisms inhabiting anoxic environments, such as certain anaerobic protists and bacteria [38, 39], which have developed specialized ion transport systems to cope with stress conditions. Organelle organization and stress responses are critical for maintaining cellular homeostasis under stress conditions or when managing the metabolic demands of symbiosis [40]. The enrichment of these processes in *T. utriculariae* suggests that this species has evolved mechanisms to regulate acidic microenvironments within its cellular compartments, such as the symbiosome that harbors the endosymbionts [40]. The expansions of genes involved in biotic interactions, ion transport, protein modification, and stress response pathways highlighted *T. utriculariae*’s need for metabolic flexibility and robust cellular regulation, likely due to the challenges of maintaining a stable symbiotic relationship.

*T. utriculariae* experienced 492 gene-family contractions and completely lost 324 of those families; in contrast, these families are present in *T. thermophila* and *T. malaccensis*. Functional analysis of the contracted gene families revealed enrichment for GO terms associated with developmental and reproductive processes (Supplementary Fig. S2, Supplementary Data S2E). Additionally, “meiosis I” and “homologous recombination” (cell cycle/DNA metabolism categories) and “cell adhesion” (cell signaling category) were overrepresented among the contracted gene families. The loss of meiosis-related genes potentially indicates that *T. utriculariae* relies on a streamlined set of core proteins for sexual reproduction or displays reduced sexual reproduction. Similarly, having fewer cell adhesion genes may lead to a reduction in cell–cell interactions, which could be involved in cell mating. Gene family contractions in core processes can reflect evolutionary specialization. For instance, other studies have shown that loss of redundant genes in essential pathways can accompany niche adaptation and speciation​ [41]. Thus, the contracted GO categories implied that *T. utriculariae* has pared down redundancy in specific fundamental pathways (e.g. sexual cell division and adhesion) for species-specific adaptation.

### *T. utriculariae* mitochondrial genome evolution reflects its adaptations to unique ecological and symbiotic pressures

**Figure 2:**
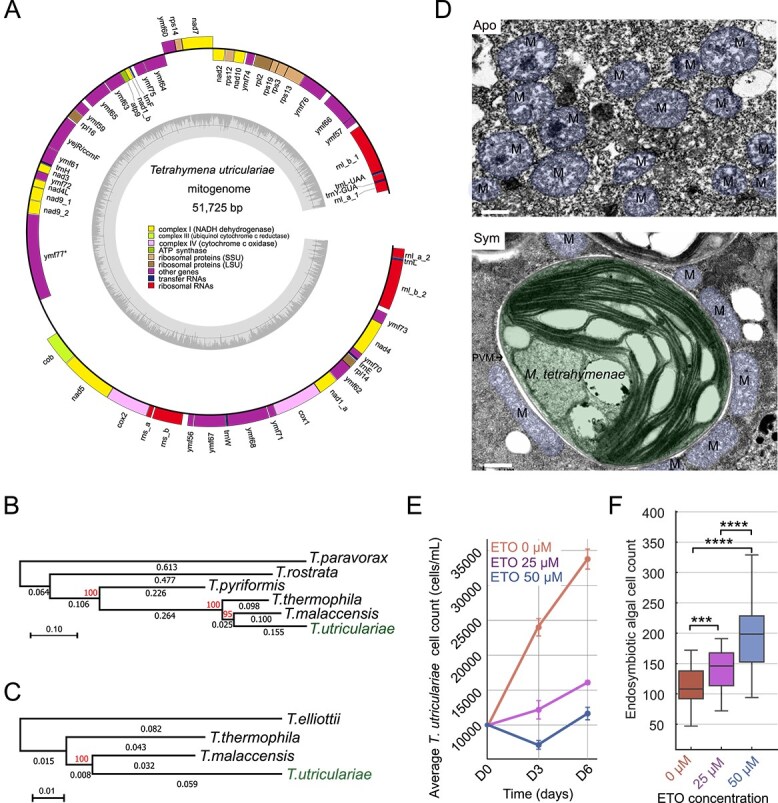
Mitochondria of *T. utriculariae* display a unique evolutionary trajectory specific to its symbiotic lifestyle. (A) Linear mitochondrial genome (51 725 bp) containing 44 protein-coding genes, seven tRNAs, and six rRNAs; genes are color-coded by function (legend in A). The inner track shows GC content; an asterisk indicates an intron-containing gene ymf77. (B) Mitochondrial-encoded proteins: maximum-likelihood tree (42 concatenated genes; IQ-TREE v3.0.1; 1000 ultrafast bootstraps). *T. utriculariae* branches are 1.55 times and 1.58 times longer than those of *T. malaccensis* and *T. thermophila*; clade support = 95%. Scale bar: 0.10 substitutions per site. (C) Nuclear-encoded mitochondrial proteins: maximum-likelihood tree (494 concatenated proteins; same settings). *T. utriculariae* branches are 1.84 times and 1.37 times longer than those of *T. malaccensis* and *T. thermophila*; clade support = 100%. Scale bar: 0.01 substitutions per site. (D) TEM images: aposymbiotic cells show rounded mitochondria (M); symbiotic cells show elongated mitochondria closely associated with the perialgal vacuole membrane (PVM) around *M. tetrahymenae*. Pseudocolor: mitochondria in blue shades; *M. tetrahymenae* in green shades. White lines are scale bars: 0.5 μm. (E) Etomoxir (ETO; 0, 25, 50 μM) reduces host growth over 6 days (*n* = 3; mean ± SEM). Statistical analysis: one-way ANOVA with Tukey *post hoc*. (F) ETO (25, 50 μM) increases endosymbiont numbers after 6 days (50 cells per sample). Statistical analysis: one-way ANOVA with Tukey *post hoc*; ^***^P < .001, ^****^P < .0001.

The mitochondrial genome of *T. utriculariae* was assembled with a total size of 51 725 bp, which is larger than the respective genomes of related species such as *T. thermophila* and *T. malaccensis* (Fig. 2A and Supplementary Table S2). The gene content includes 44 protein-coding genes, seven tRNA genes, and six rRNA genes, with a larger intergenic region (6534 bp) compared to its relatives and an A + T content of 78.54%.

Maximum likelihood phylogenies were generated from the concatenated alignments of mitochondrial DNA-encoded proteins (Fig. 2B) and nuclear-encoded mitochondrial proteins (Fig. 2C) using IQ-TREE v3.0.1 with 1000 ultrafast bootstrap replicates (Supplementary Data S2A and B). The mitochondrial gene phylogeny was aligned with the whole-genome tree (Fig. 1B), showing 95% bootstrap support for the *T. malaccensis* and *T. utriculariae* clade and 100% support for deeper nodes. Quantitatively, the *T. utriculariae* mitochondrial branch length (0.155 substitutions per site) was 1.55 times and 15.8 times longer than those of *T. malaccensis* (0.100) and *T. thermophila* (0.098), respectively. Tajima’s relative rate tests [42] confirmed this acceleration, rejected equal evolutionary rates in both reciprocal outgroup comparisons (*T. thermophila* outgroup: χ^2^ = 95.55, df = 1, *P* < .0001; *T. malaccensis* outgroup: χ^2^ = 72.16, df = 1, *P* < .0001; Supplementary Table S3). Because mitochondrial-encoded proteins function in concert with nuclear-encoded mitochondrial proteins, we assessed whether the nuclear-encoded mitochondrial proteins co-evolved to exhibit a similar pattern. We determined that the nuclear-encoded mitochondrial genes in *T. utriculariae* also changed more rapidly than those of *T. malaccensis* and *T. thermophila* (Fig. 2C). The nuclear-encoded mitochondrial protein phylogeny showed 100% bootstrap support for the (*T. malaccensis*, *T. utriculariae*) clade, with *T. utriculariae* again exhibiting rate acceleration. Quantitatively, the *T. utriculariae* branch length (0.059 substitutions per site) was 1.84 times and 1.37 times longer than those of *T. malaccensis* (0.032) and *T. thermophila* (0.043), respectively. Tajima’s relative rate tests also rejected equal rates in both reciprocal outgroup comparisons (*T. thermophila* outgroup: χ^2^ = 784.86, df = 1, *P* < .0001; *T. malaccensis* outgroup: χ^2^ = 141.08, df = 1, *P* < .0001; Supplementary Table S3). Together, these measurements demonstrated a coordinated evolutionary acceleration across mitochondrial machinery in *T. utriculariae.*

Transmission electron microscopy (TEM) on *T. utriculariae* mitochondria revealed that they are remodeled in symbiotic cells. Aposymbiotic *T. utriculariae* cells predominantly hosted rounded mitochondria (Fig. 2D and Supplementary Fig. S4A). In contrast, the mitochondria of symbiotic cells were elongated and intimately associated with the membrane of the perialgal vacuole (PV), which houses the endosymbiont; most nonelongated mitochondria were not in contact with the PV membrane (Fig. 2D, Supplementary Fig. S4B and C). The elongated shape and proximity of mitochondria to the PV may facilitate efficient energy transfer, potential metabolite exchange, and redox homeostasis at the host–symbiont interface. Alternatively, as shown in a recent study on the parasitic *Toxoplasma gondii*, mitochondrial wrapping can sequester host fatty acids, thereby limiting nutrient availability to the parasite and controlling its proliferation [43]. This scenario raises the possibility that mitochondrial remodeling in *T. utriculariae* may also contribute to regulating the endosymbiont population.

Symbiotic cells were treated with etomoxir to inhibit fatty acid oxidation in mitochondria, aiming to examine the regulatory role of fatty acid oxidation and mitochondria in photoendosymbiosis. Etomoxir is an irreversible inhibitor of carnitine palmitoyltransferase 1 (CPT1), a rate-limiting enzyme in mitochondrial fatty acid oxidation [44]. Treatment of symbiotic *T. utriculariae* cells with etomoxir resulted in a dose-dependent reduction in host growth rates (Fig. 2E), supporting that mitochondrial fatty acid oxidation is crucial for sustaining host cell growth and metabolic activity. However, inhibition of host fatty acid oxidation also significantly increased the number of endosymbionts within the host cells (Fig. 2F), indicating that mitochondrial fatty acid oxidation also acts as a mechanism to regulate endosymbiont numbers. This specific regulatory mechanism in *T. utriculariae* may explain the unique evolutionary pattern of its mitochondrial genes. We acknowledge that genetic perturbation approaches (e.g. RNAi or CRISPR) would provide complementary evidence for this mechanism, but such tools are not yet established for *T. utriculariae*. Future development of genetic manipulation techniques for this organism would strengthen these conclusions.

### Transcriptomic analysis reveals dynamic host responses to symbiosis and environmental conditions

We conducted a time-series RNA-seq experiment comparing symbiotic (Sym) and aposymbiotic (Apo) *T. utriculariae* cells at six time points (AM8, AM11, PM2, PM5, PM8, PM11) through a single 24-h light–dark cycle (Supplementary Data S3A). This approach allowed us to capture temporal changes in gene expression associated with symbiosis and environmental cues.

**Figure 3:**
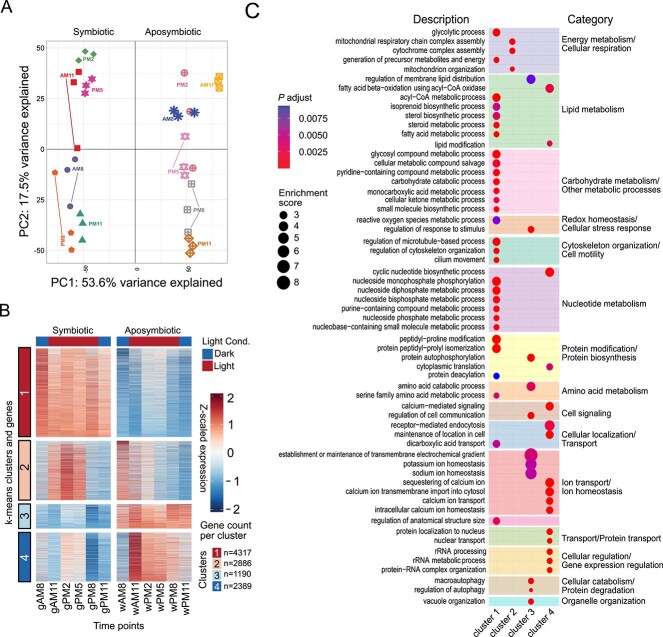
Transcriptomic dynamics of *T. utriculariae* reveal distinct metabolic signatures in symbiotic and aposymbiotic states across diurnal cycles. (A) Principal component analysis (PCA) of transcriptomes from symbiotic (Sym, solid symbols) and aposymbiotic (Apo, hollow symbols) cells sampled at six time points; PC1 (53.6%) separates symbiotic status, and PC2 (17.5%) captures time-of-day variation. (B) *K*-means clustering of differentially expressed genes (*n* = 6 time points per condition) resolved four clusters: 1 (4317 genes) upregulated in Sym; 2 (2886) peaking at midday in Sym; 3 (1190) upregulated in Apo; and 4 (2389) with morning expression in Apo. (C) Gene ontology enrichment for each cluster; circle size reflects enrichment score, and color intensity reflects the adjusted *P*-value.

A principal component analysis (PCA) of the transcriptomic data revealed that symbiotic cells clustered distinctly from aposymbiotic cells, indicating that the symbiotic state is the primary factor influencing gene expression patterns, accounting for 53.6% of the total variance along the first principal component (PC1) (Fig. 3A). Light conditions also contributed to varied gene expression within symbiotic cells, as samples collected during light periods (AM11, PM2, PM5) were well separated from those collected in the transition to or during dark periods (AM8, PM8, PM11), underscoring the combined influence of symbiosis and light on the host’s transcriptional landscape.

We defined a gene as being differentially expressed if expression in symbiotic cells was 2-fold higher or lower than that in aposymbiotic cells (adjusted *P*-value <.01, Student’s *t*-test with Benjamini–Hochberg correction) at any time point (Supplementary Data S3B). Using this cutoff, we identified 10 782 differentially expressed genes that could be roughly classified into four clusters (Fig. 3B).

One prominent set of genes (Cluster 1, *n* = 4317) exhibited stable expression in symbiotic cells across all time-points, but were downregulated in aposymbiotic cells (Fig. 3B, Supplementary Data S3C). GO analysis revealed that these genes are involved in energy, lipid, and carbohydrate metabolism, as well as homeostasis and cytoskeleton organization (Fig. 3C, Supplementary Data S3D). Genes associated with energy generation were consistently expressed, indicating host reliance on efficient energy production, possibly to meet the metabolic demands of both the host and the symbiont [45]. Given that our etomoxir treatment experiments revealed that *T. utriculariae* cells adjust fatty acid oxidation pathways to control the endosymbiont population (Fig. 2F), it may explain why lipid metabolism pathways are upregulated in symbiotic cells. Furthermore, the host may actively contribute to the nutritional needs of its endosymbionts, supplying key metabolites to sustain the symbiotic balance [46]. The enrichment for genes linked to cytoskeleton organization and microtubule-based movement indicates that these genes are involved in maintaining the cellular structures necessary for housing endosymbionts, potentially stabilizing the perialgal vacuole in which the algae reside [47, 48]. These core metabolic and structural processes appear to be essential for the continuous support of the endosymbiont, irrespective of external light conditions, underscoring the stable and foundational role of this gene set in symbiotic maintenance.

Cluster 2, comprising 2886 genes, showed increased expression in symbiotic cells during midday light conditions (Fig. 3B, Supplementary Data S3C). These genes were enriched in mitochondrial respiratory chain complex assembly and organization (Fig. 3C, Supplementary Data S3D). Upregulation of mitochondrial genes during light exposure indicates coordination between host mitochondrial function and the endosymbiont’s photosynthetic activity [49, 50]. Together with the observation that mitochondria-related genes in *T. utriculariae* evolve more rapidly (Fig. 2B and C) and that mitochondrial functions contribute to regulating endosymbiont numbers (Fig. 2F), this outcome illustrates the significance of host mitochondrial functions in endosymbiosis.

Cluster 3, comprising 1190 genes, was significantly upregulated in aposymbiotic cells. GO analysis revealed enrichment for autophagy-related processes, vacuole organization, and ion homeostasis (Fig. 3C, Supplementary Data S3D). The induction of autophagy indicates that aposymbiotic cells activate internal recycling mechanisms in the absence of endosymbionts, repurposing cellular components to maintain homeostasis [51, 52]. The upregulation of genes associated with vacuole organization further supports the adaptation of intracellular structures for nutrient management.

Another set of genes (Cluster 4, *n* = 2389) exhibited high expression during early light exposure in aposymbiotic cells, highlighting the host’s ability to respond to light cues even in the absence of the endosymbiont. These genes were significantly enriched in processes related to calcium ion homeostasis, nuclear transport, and ribosomal RNA (rRNA) metabolism. Although it remains unclear why aposymbiotic cells need to alter the expression of these genes under these particular conditions, our data indicate that aposymbiotic cells are highly responsive to environmental light, adjusting their physiology to optimize cellular functions.

Collectively, our findings from gene expression analysis demonstrate that *T. utriculariae* orchestrates a complex transcriptional response to balance symbiotic maintenance with different survival strategies, which are modulated by both the symbiotic state and environmental factors such as light.

### Endosymbiont *M. tetrahymenae* exhibits metabolic shifts during symbiosis

**Figure 4:**
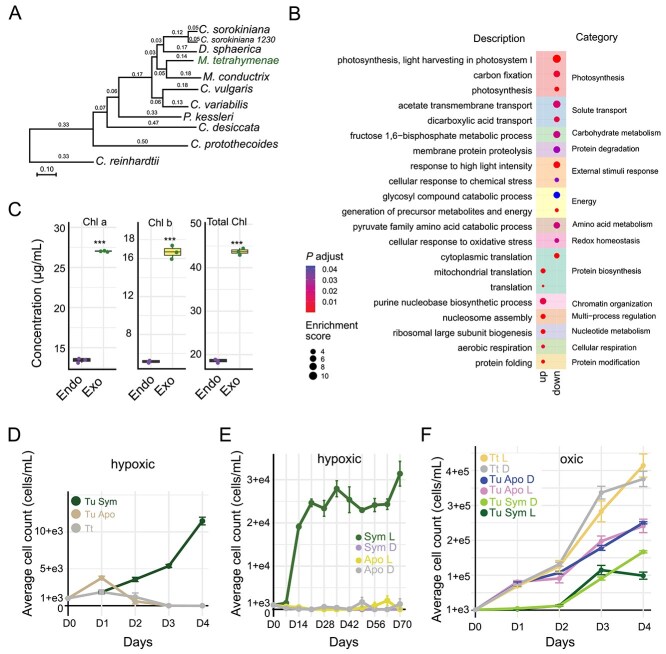
Transcriptomic and physiological responses of *M. tetrahymenae* in symbiotic and free-living states. (A) Maximum-likelihood phylogeny of chlorophytes showing the position of *M. tetrahymenae* (green label); branch lengths indicate genetic distance (scale bar: 0.10 substitutions per site). (B) Gene Ontology enrichment for genes differentially expressed in endosymbiotic versus free-living *M. tetrahymenae*; point size denotes enrichment score and color encodes adjusted *P*-value. (C) Chlorophyll a, chlorophyll b, and total chlorophyll are lower in endosymbiotic than free-living algae; ^***^P < .001 (two-tailed Student’s *t*-test). (D) Short-term hypoxia: growth curves for symbiotic *T. utriculariae* (Tu Sym), aposymbiotic *T. utriculariae* (Tu Apo), and *T. thermophila* (Tt); mean ± SEM (*n* = 3). (E) Long-term hypoxia: cell counts for Tu Sym in light (Sym L) and dark (Sym D) and Tu Apo in light (Apo L) and dark (Apo D) over 70 days; Tu Sym L reached ~32 000 cells/ml by day 70; mean ± SEM (*n* = 3). (F) Oxic conditions: growth of Tu Sym and Tu Apo under light–dark cycles (L) or constant dark (D), with Tt controls; mean ± SEM (i = 3).

To understand the symbiotic relationship from the endosymbiont’s perspective, we sequenced and annotated the genome of *M. tetrahymenae* using a hybrid assembly approach. We combined Illumina paired-end short reads for accuracy and Oxford Nanopore long reads for contiguity. The assembly was polished iteratively with both data types, resulting in a high-quality genome characterized by a size of 66.4 Mb, a guanine–cytosine (GC) content of 65.29%, and 97.1% BUSCO completeness (Table 2). Annotation of the nuclear genome was performed by incorporating RNA-seq data to enhance gene model predictions, leading to the identification of 14 229 protein-coding genes. A one-to-one orthologous gene-based maximum likelihood phylogenetic tree clustered *M. tetrahymenae* with *M. conductrix* (Fig. 4A). Additionally, we assembled the plastid and mitochondrial genomes (Supplementary Fig. S5A and B), which displayed sizes of 133 089 bp and 91 655 bp, respectively, encoding 79 and 32 protein-coding genes (Table 2). Average protein lengths for the nuclear, plastid, and mitochondrial genomes are 478, 265, and 262 amino acids, respectively (Table 2).

**Table 2:** Genomic features of the *M. tetrahymenae* nuclear, plastid, and mitochondrial genomes.

Genome type	Total assembled scaffolds/ contigs	Genome size (Mb)	N50 (Mb)	Shortest protein-coding sequence (bp)	Longest protein-coding sequence (bp)	Genomic GC content (%)	Gene number	Average length of protein-coding sequence (bp)	Median length of protein-coding sequence (bp)	BUSCO completeness (%)
Nuclear genome	67	66.4	3.29	111	30 000	65.29	14 229	1434	1134	97.1
Plastid genome		0.133		96	4470	34.22	79	794	483	
Mitochondrial genome		0.0917		225	2019	30.25	32	787	651	

We analyzed the transcriptomic profiles of *M. tetrahymenae* in its endosymbiotic (Endo) and free-living (Exo) states, as collected at midday (2 p.m.) (Supplementary Data S4A). Differential expression analysis identified 1388 genes significantly upregulated and 845 genes significantly downregulated (fold-change >2, adjusted *P*-value <.01, Student’s *t*-test with Benjamini–Hochberg correction) in endosymbiotic *M. tetrahymenae* compared to free-living cells (Supplementary Data S4B). Genes involved in protein biosynthesis, nucleotide biosynthesis, and aerobic respiration were upregulated in the endosymbiotic *M. tetrahymenae* (Supplementary Data S4C), suggesting increased metabolic and biosynthetic activities. This shift toward enhanced metabolic processes supports that the endosymbiotic *M. tetrahymenae* cells can thrive inside the host.

Genes associated with GO terms related to photosynthesis and light-harvesting complexes, such as light harvesting in “photosystem I” (GO:0009768), as well as carbon fixation (GO:0015977), were downregulated in the endosymbiotic state (Fig. 4B, Supplementary Data S4C), indicating reduced photosynthetic activity by the endosymbiont inside the host. The identification of GO categories enriched for dicarboxylate and succinate transport processes (GO:0015740, GO:0006835) further suggests a potential metabolic shift by endosymbionts toward reduced photosynthesis (Fig. 4B, Supplementary Data S4C). How can endosymbiotic algal cells support these increased metabolic and biosynthetic activities when their levels of photosynthesis are decreased? Our etomoxir treatment experiments (Fig. 2F), combined with transcriptomic evidence of reduced photosynthetic gene expression and increased transport processes, suggest that the endosymbionts may rely more on nutrients from the host, consistent with a potential transition toward a heterotrophic lifestyle.

To further explore the effect of reduced photosynthetic gene expression by endosymbionts, we measured the chlorophyll content of endosymbiotic and free-living algal cells, which is a vital light-absorbing component of photosynthesis. Endosymbiotic *M. tetrahymenae* exhibited significantly lower levels of chlorophyll a, b, and total chlorophyll compared to free-living cells (Fig. 4C). Together, these results indicate that the photosynthetic capabilities of endosymbionts are diminished within the host environment. The endosymbionts likely acquire nutrients from the host to compensate for their reduced production of photosynthetic products, raising the possibility of parasitic interactions. Whereas our transcriptomic evidence of reduced photosynthetic gene expression combined with direct measurements of decreased chlorophyll content strongly supports a metabolic shift toward reduced autotrophy in endosymbiotic *M. tetrahymenae*, direct evidence for nutrient uptake or metabolite exchange via stable isotope labeling or metabolomics experiments would further strengthen conclusions about the extent and mechanisms of host–symbiont nutrient transfer.

### Symbiosis imposes a metabolic burden on *T. utriculariae*

*Tetrahymena utriculariae* was originally discovered inside the traps of *U. reflexa*, where oxygen levels are low due to the decomposition of organic matter and limited gas exchange [53, 54]. It has been suggested that the endosymbiotic *M. tetrahymenae* provides essential oxygen to support host growth under this hypoxic condition [15, 54]. Consequently, we cultured both symbiotic and aposymbiotic *T. utriculariae* cells under artificial hypoxic conditions and monitored them for both short (4 days) and long (70 days) durations (Fig. 4D and E). Only symbiotic cells under a light–dark cycle exhibited robust and sustained proliferation, maintaining a high cell density throughout both experimental periods. In contrast, symbiotic cells under constant dark or aposymbiotic cells showed minimal to no growth (Fig. 4E), indicating that whereas the endosymbiont provides a survival advantage to the host under hypoxic conditions, this benefit depends entirely on light exposure.

We observed a different growth pattern when *T. utriculariae* cells were cultured in oxic conditions. Aposymbiotic cells exhibited higher growth rates than symbiotic cells, regardless of whether under light–dark or constant-dark conditions (Fig. 4F). This result suggests that when oxygen is not a limiting factor, the endosymbiotic relationship imposes a metabolic cost on the host. The photosynthetic activity of the endosymbionts cannot alleviate the cost. This outcome is consistent with our endosymbiont transcriptome data, which showed that *M. tetrahymenae* inside *T. utricularia* exhibits signatures suggestive of a shift from autotrophy to mixotrophy. It is likely that endosymbiotic *M. tetrahymenae* also imposes similar costs under hypoxic conditions. However, the benefit of the oxygen supply exceeds the metabolic burden, allowing symbiotic *T. utriculariae* to proliferate (Fig. 4D and E)*.* The parasitic aspect of endosymbiotic *M. tetrahymenae* may explain why the host needs to evolve specific mechanisms involving mitochondria remodeling and fatty acid oxidation to control the endosymbiont population (Fig. 2).

Collectively, our findings indicate that the photoendosymbiotic association between *T. utriculariae* and *M. tetrahymenae* is highly dependent on environmental conditions. Although *M. tetrahymenae* limits host proliferation in oxic environments, it provides a clear survival benefit under long-term hypoxic conditions. This scenario strongly contrasts with the stable relationship between *P. bursaria* and *Chlorella* spp., in which aposymbiotic cells consistently exhibited lower fitness than symbiotic cells in all tested conditions [55]. Thus, our findings highlight the complex interplay between mutualism and parasitism at the early stages of endosymbiosis evolution.

## Discussion

The symbiotic relationship between the freshwater ciliate *T. utriculariae* and its algal endosymbiont *M. tetrahymenae* offers a unique model for studying the early stages of photoendosymbiosis. Our integrative analysis revealed that this association deviates from classical mutualistic photoendosymbiosis, instead being characterized by conditional benefits and significant fitness costs to the host. Our findings provide insights into the incipient evolutionary stage of photoendosymbiosis, i.e. before a stable relationship is established.

Multiple lines of evidence from our analysis support that the mitochondria of *T. utriculariae* have evolved specifically to support its unique lifestyle. We observed fast-evolving patterns in mitochondria-related genes and elongated mitochondria closely associated with endosymbionts (Fig. 2B–D and Supplementary Fig. S4B). Moreover, the genes involved in mitochondrial organization and respiration are significantly upregulated in symbiotic host cells (Fig. 3C). These adaptations may reflect a shift towards anaerobic metabolism or enhanced efficiency under low-oxygen conditions, as previously suggested for other anaerobic eukaryotes such as *Blastocystis* spp. and certain ciliates [56, 57]. In addition, prior work shows that pathogen-containing compartments often form membrane contact sites with host organelles, including mitochondria [43, 58, 59], and that pathogens can proliferate within these compartments [60–62]. Consistent with this, our experiments revealed a role for mitochondrial fatty acid oxidation in regulating the proliferation of endosymbionts. When we reduced host mitochondrial fatty acid oxidation using etomoxir, *T. utriculariae* cells displayed decreased growth but increased numbers of endosymbionts (Fig. 2E and F). In *To. gondii*, enhanced host mitochondrial fatty acid oxidation limits parasite access to fatty acids and restricts parasite growth [43], suggesting a convergent principle of host metabolic control. By analogy, *T. utriculariae* may use mitochondrial fatty acid oxidation as a host-side control to limit algal proliferation and thereby reduce the metabolic burden imposed by the endosymbiont [43].

A recent study analyzed gene expansions in the *T. utriculariae* and *M. tetrahymenae* genomes [63]. Their results indicate that gene families associated with stress response have been expanded in both the host and endosymbiont as their photoendosymbiotic relationship evolved, supporting the notion that the early stage of endosymbiosis is stressful and requires adaptation in both partners. This stress-driven adaptation aligns with the experimental evolution studies, which have shown that laboratory-induced symbioses exhibit rapid physiological adjustments in both partners [25, 26].

Environmental context governs the outcome (mutualistic vs. parasitic) and the stability of early endosymbiosis. Across protists, the condition-dependent nature of symbiosis is evident in diverse protist systems. For example, the mixotrophic ciliate *Stentor pyriformis* retains *Chlorella* spp. as endosymbionts and stores starch in extremely oligotrophic habitats, an ecological constraint tightly linked to symbiont maintenance [64]. In amoebozoan photosymbiosis, *Mayorella viridis* exhibits comparable context-dependence, being dispensable under nutrient-rich conditions but beneficial during starvation [7]. In anaerobic marine ciliates, obligate endosymbionts perform denitrification to supply host energy, a solution that only makes sense under persistent anoxia [65]. Comparable environment-defined constraints are also widely documented in metazoans that rely on chemosynthetic endosymbionts in vents and seeps [66]. Such conditional outcomes and shifting fitness balances align with the parasite–mutualist continuum, which proposes that symbioses can move along an antagonism–mutualism axis depending on ecological context [67]. Spatially explicit models further indicate that local (vertical-like) transmission and spatial structure can drive the emergence and stabilization of mutualism from initially parasitic interactions [68].

It is possible that the ancestors of *M. tetrahymenae* survived passage through the digestive system of *T. utriculariae* owing to cell-wall properties that confer resistance to lysosomal degradation. In *P. bursaria*, for example, symbiotic *Chlorella* spp. do not block acidification or lysosomal fusion of host digestive vacuoles during infection, and they can create temporary resistance to host lysosomal enzymes in early stages of infection, illustrating that intracellular algae can persist despite the digestive response [18, 69]. A mechanistically analogous strategy also exists in intracellular parasites that exclude lysosomal fusion altogether (e.g. *Toxoplasma gondii*), underscoring how intracellular residents can avoid destruction long enough to exploit host resources [70]. After avoiding lysosomal degradation, the algae would be exposed to a nutrient-rich intracellular environment that can favor their proliferation and exploitation of the host. Living inside the host cell would also be expected to reduce contact with viruses that infect algae [71]; chloroviruses, for example, readily infect free-living *Chlorella variabilis*, whereas algae enclosed within host compartments are less accessible to these viruses [72], providing an additional selective advantage for maintaining the endosymbiotic lifestyle despite its parasitic costs to the host under oxic conditions.

Parasite-like algal cells could kill the host if they proliferated unchecked, forcing the host cells to evolve a mechanism to control the algal population in their cytoplasm. Nevertheless, because of their reduced fitness, infected ciliate cells are more likely to be outcompeted by uninfected cells and thus lost from the population. By contrast, the low-oxygen environment inside *U. reflexa* traps represents a critical turning point in the evolution of the endosymbiotic relationship, as only infected *T. utriculariae* cells can propagate and exploit this nutrient-rich hypoxic environment. Because *Utricularia* traps constantly grow and die, infected *T. utriculariae* cells must migrate and thrive under both hypoxic and oxic conditions. If such conditions persist for a long time, it will further select for *T. utriculariae* and *M. tetrahymenae* cells to co-evolve an adjusted physiology that reduces costs and increases benefits, driving the evolution of a stable endosymbiotic relationship.

## Supplementary Material

ISMEJ_D_25_01009R2_Supplementary_Figures_and_Tables_wraf203

Supplementary_Data_S1_wraf203

Supplementary_Data_S2_wraf203

Supplementary_Data_S3_wraf203

Supplementary_Data_S4_wraf203

ISMEJ-D-25-01009R2_Supplementary_text_wraf203

## Data Availability

The polished genome, gene annotation, CDS sequences, and protein sequences have all been uploaded to NCBI (*T. utriculariae*: PRJNA1236653, *M. tetrahymenae*: PRJNA1243065). Gene annotations and genomic data are available from the *Tetrahymena* Genome Database (tet.ciliate.org). Genome and RNA sequencing data from the current work have been deposited in the NCBI database (*T. utriculariae*: PRJNA1236653; *M. tetrahymenae*: PRJNA1243065). All the other data are available in the main text or the supplementary materials. Scripts used for data analysis and plotting have been uploaded to Figshare (https://doi.org/10.6084/m9.figshare.29939144.v5).
