# Supplementary material for: Environment-dependent mutualism–parasitism transitions in the incipient symbiosis between Tetrahymena utriculariae and Micractinium tetrahymenae
Source: ISME J. 2025 Sep 6;19(1):wraf203. doi: 10.1093/ismejo/wraf203 (PMC12516953; doi:10.1093/ismejo/wraf203)
Supplement: ISMEJ_D_25_01009R2_Supplementary_Figures_and_Tables_wraf203 [file ismej_d_25_01009r2_supplementary_figures_and_tables_wraf203.pdf]

Environment-dependent mutualism-parasitism transitions in the incipient symbiosis between *Tetrahymena utriculariae* and *Micractinium tetrahymenae*

Kamal Md Mostafa[1#], Yu-Hsuan Cheng[1,†,#], Li-Wen Chu[1], Phuong-Thao Nguyen [1,2], Chien-Fu Jeff Liu[1], Chia-Wei Liao[1], Thomas Posch[3], Jun-Yi Leu[1,*]

[1] Institute of Molecular Biology, Academia Sinica, Taipei, 11529, Taiwan

[2] Department of Life Sciences, National Central University, Taoyuan, 320, Taiwan

[3] Limnological Station, Department of Plant and Microbial Biology, University of Zurich, Kilchberg, Switzerland

[#] These authors contributed equally.

[†] Present address: Morgridge Institute for Research, University of Wisconsin-Madison, Madison, WI, USA

[*] Corresponding author: Jun-Yi Leu, Institute of Molecular Biology, Academia Sinica, 128 Sec. 2, Academia Road, Taipei 115, Taiwan. Email: jleu@as.edu.tw

This file contains Supplementary Figures and Tables

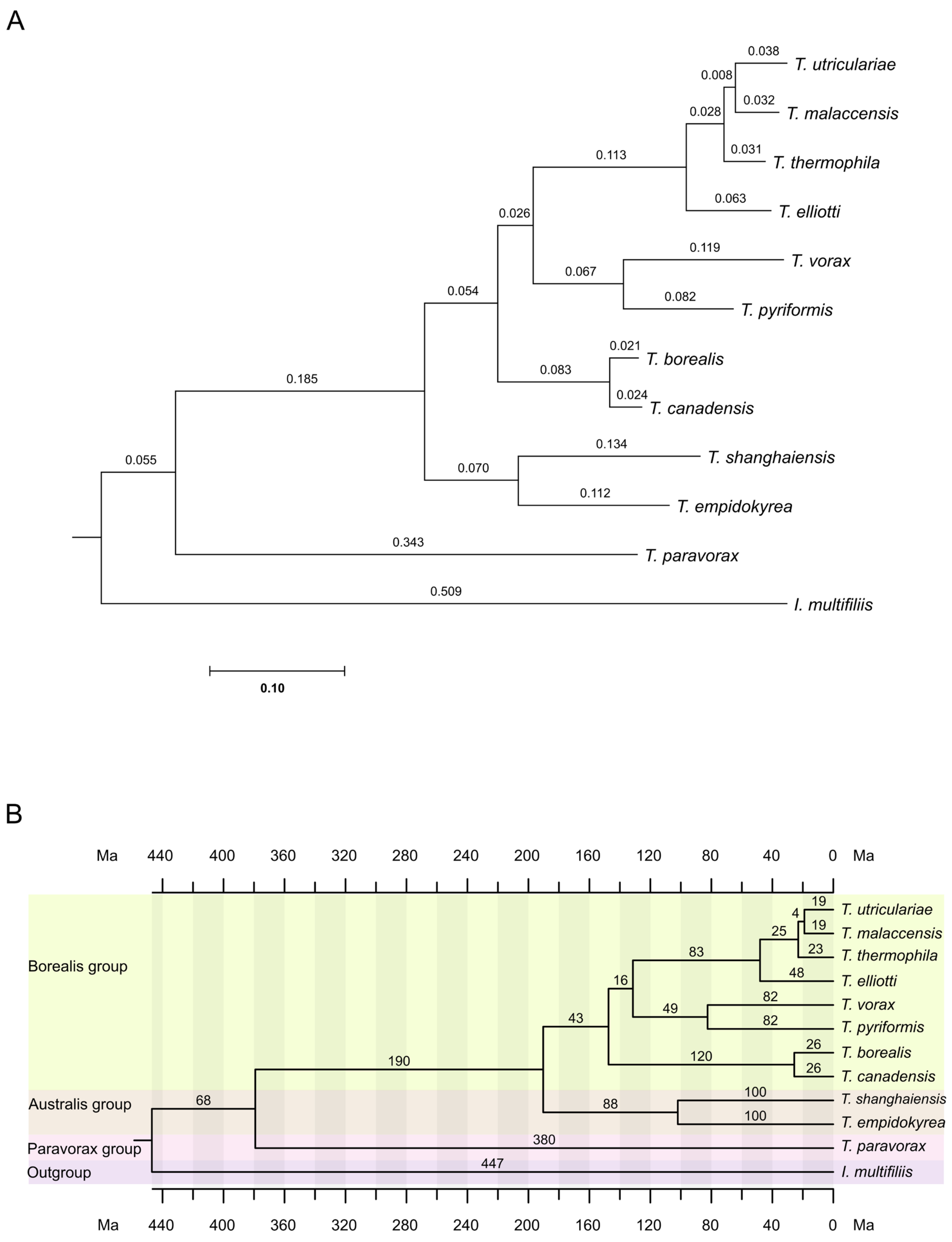

Supplementary Figure S1: Phylogenetic relationships and evolutionary timeline of *Tetrahymena* species.

(A) Maximum-likelihood phylogeny for 12 *Tetrahymena* species with *Ichthyophthirius multifiliis* as outgroup, inferred from a concatenated alignment of single-copy orthologs identified with

OrthoFinder. Branch lengths indicate genetic distance (scale bar: 0.10 substitutions per site). *T. utriculariae* forms a clade with *T. malaccensis* and *T. thermophila*. (B) Time-calibrated tree showing divergence times (Ma) for major *Tetrahymena* lineages. The genus originated ~447 million years ago (Ma), with diversification occurring near 380 Ma (Paravorax divergence) and 190 Ma (Borealis-Australis split). Within Borealis, *T. utriculariae* diverged from *T. thermophila* ~23 Ma and from *T. malaccensis* ~19 Ma.

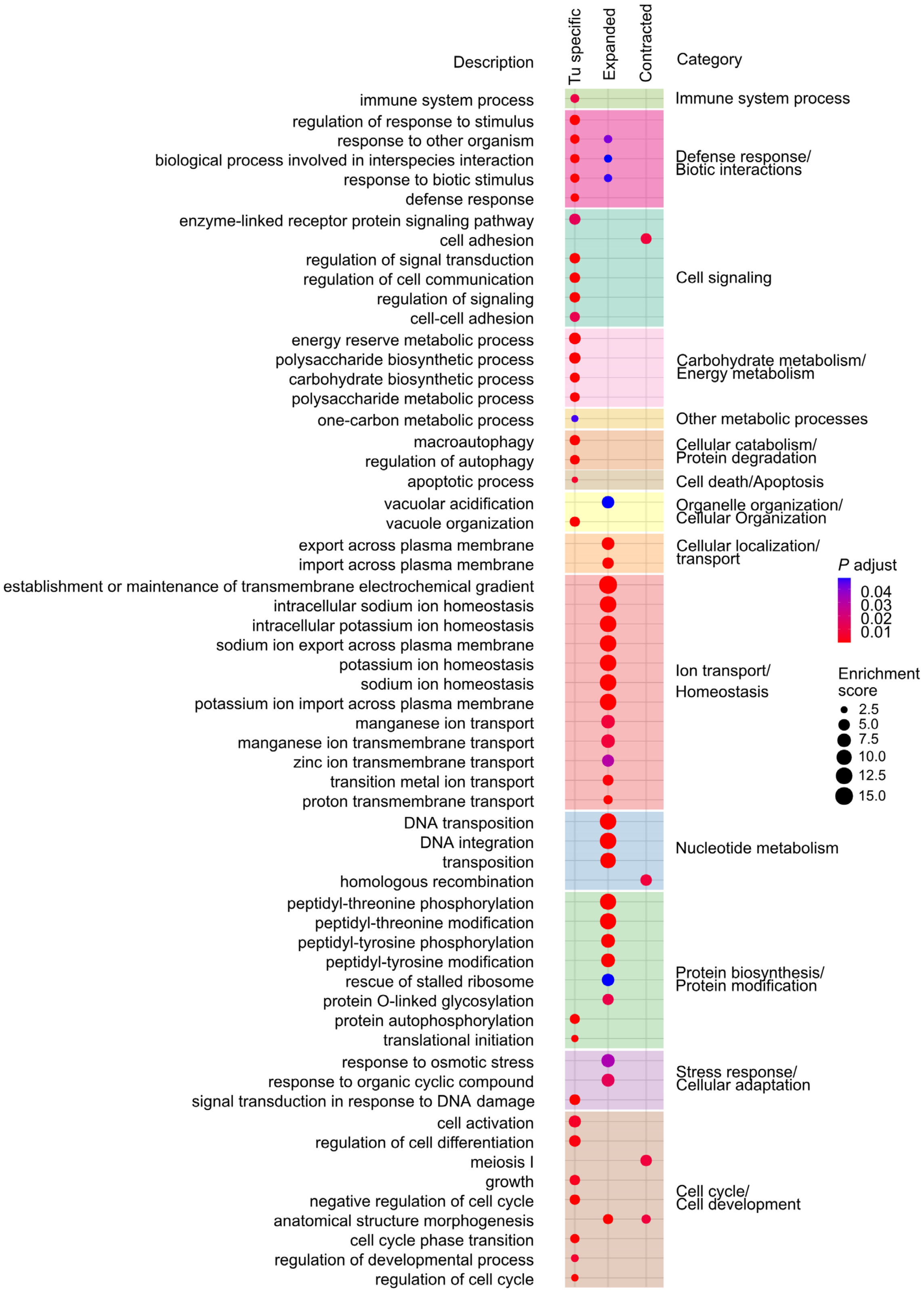

Supplementary Figure S2: GO enrichment analysis of *T. utriculariae* gene categories: *T. utriculariae*-specific, expanded, and contracted genes.

Enriched GO terms for *T. utriculariae*-specific genes, expanded gene families, and contracted gene families. *T. utriculariae*-specific and expanded sets are enriched for ion transport/homeostasis, signal

transduction, protein modification, and stress responses; contracted families are enriched for developmental processes and cell adhesion. Circle size denotes the enrichment score, and color encodes the adjusted *P* value.

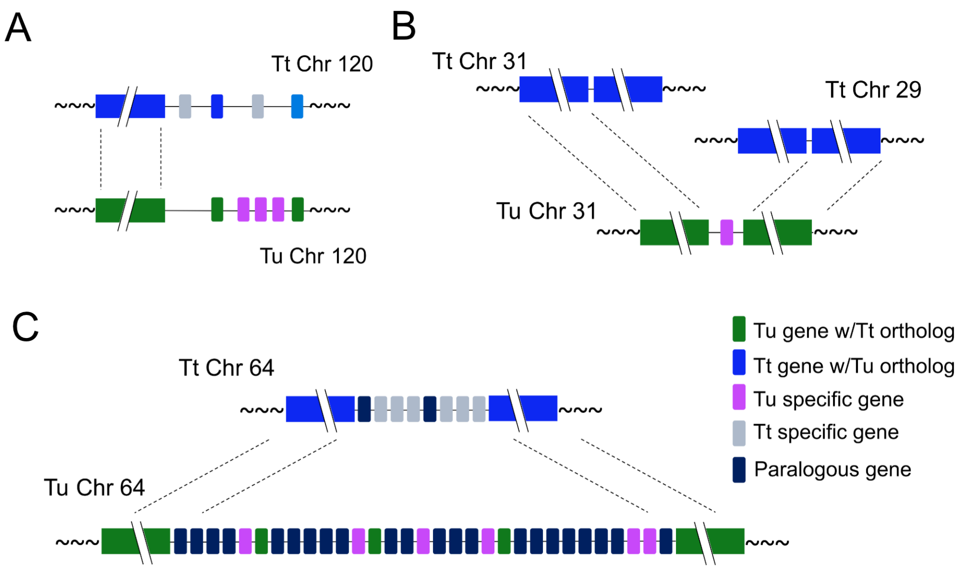

Supplementary Figure S3: Comparative genomic architecture reveals chromosomal evolution between *T. utriculariae* and *T. thermophila*.

(A) Telomeric species-specific genes: chromosome 120 comparison shows a conserved central syntenic region (green/blue blocks) and terminal *T. utriculariae*-specific genes (purple). (B) Fusion region: material from two *T. thermophila* chromosomes (Tt Chr 31 and Tt Chr 29) corresponds to a single *T. utriculariae* chromosome (Tu Chr 31); syntenic blocks in green/blue; non-syntenic species-specific genes in purple. (C) Expanded region: *T. utriculariae* chromosome 64 is longer than its *T. thermophila* counterpart; paralogous, duplication-derived genes are shown in dark blue.

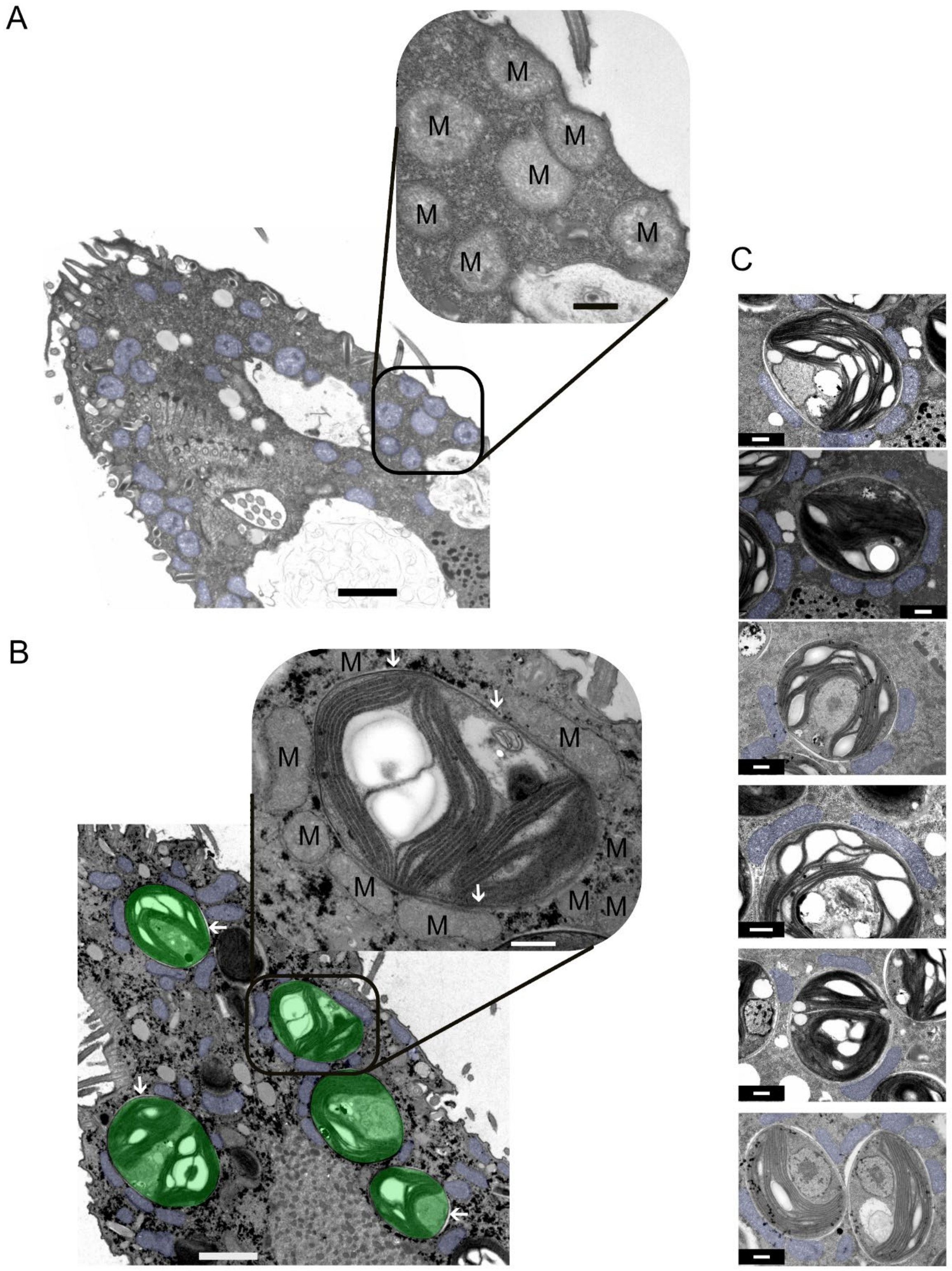

Supplementary Figure S4: Distinct mitochondrial morphologies in aposymbiotic versus symbiotic *T. utriculariae* cells. Continuation from Figure 2D.

(A) Aposymbiotic cell: mitochondria (pseudocolored blue) display rounded to oval morphologies throughout the cytoplasm. Main image scale bar: 2 µm; inset scale bar: 0.5 µm. (B) Symbiotic cell containing *M. tetrahymenae* (green pseudocolor): mitochondria (blue) are elongated and closely associated with the perialgal vacuole membrane (PVM; white arrow). Main image scale bar: 2 µm;

inset scale bar: 0.5 µm. (C) Gallery of symbiotic cells maintained under low oxygen, showing consistent mitochondria–perialgal vacuole associations; mitochondria in blue pseudocolor and endosymbionts in green. Scale bars (white line inside black box): 0.5 µm.

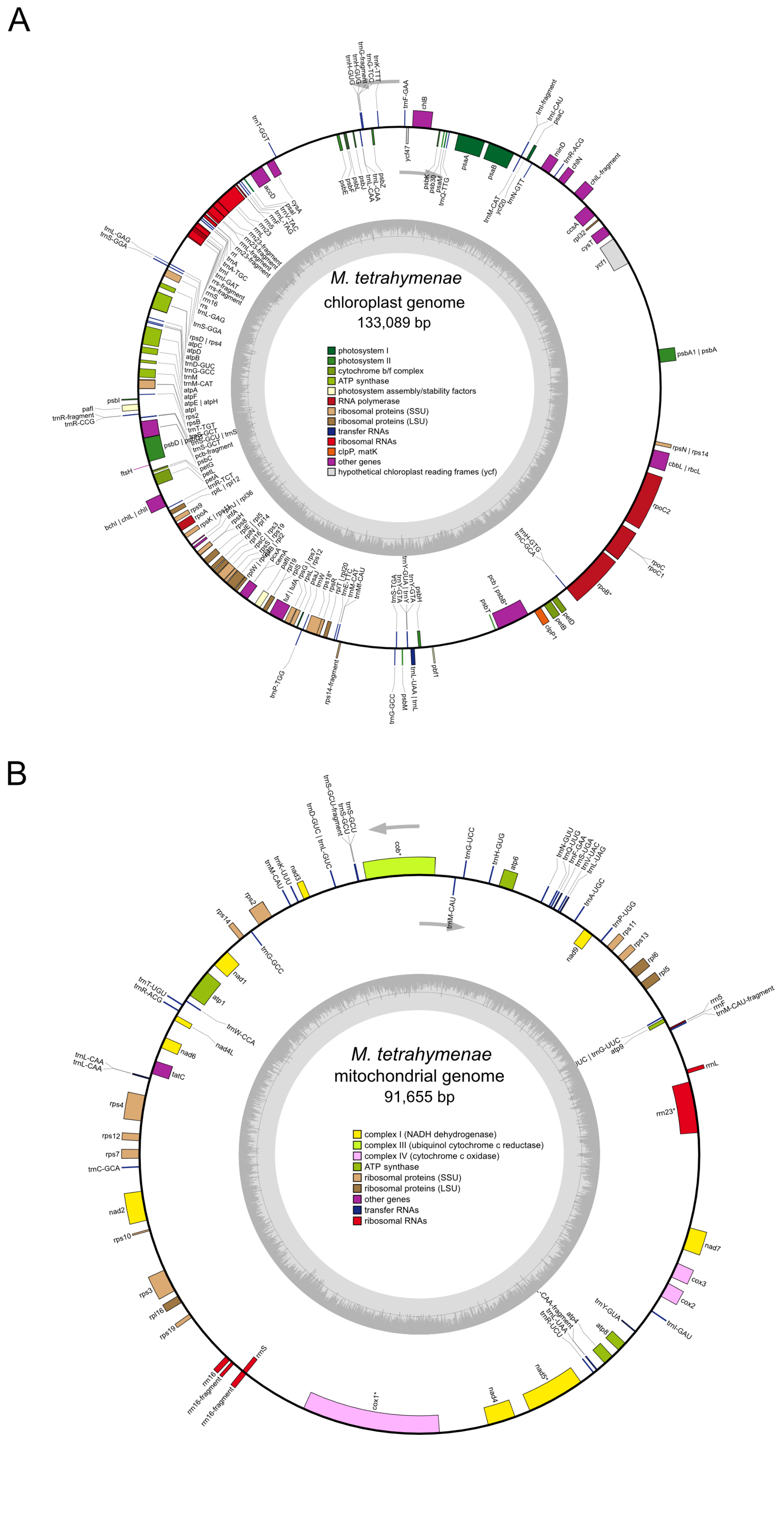
A
M. tetrahymenae
chloroplast genome
133,089 bp
photosystem I
photosystem II
cytochrome b/f complex
ATP synthase
photosystem assembly/stability factors
RNA polymerase
ribosomal proteins (SSU)
ribosomal proteins (LSU)
transfer RNAs
ribosomal RNAs
clpP, matK
other genes
hypothetical chloroplast reading frames (ycf)
B
M. tetrahymenae
mitochondrial genome
91,655 bp
complex I (NADH dehydrogenase)
complex III (ubiquinol cytochrome c reductase)
complex IV (cytochrome c oxidase)
ATP synthase
ribosomal proteins (SSU)
ribosomal proteins (LSU)
other genes
transfer RNAs
ribosomal RNAs

Supplementary Figure S5: Organellar genome architecture of the endosymbiotic alga *M. tetrahymenae*.

(A) Circular plastid genome (133,089 bp) with genes color-coded by function (photosystems I/II, cytochrome b/f, ATP synthase, assembly/stability factors, RNA polymerase, ribosomal proteins [SSU/LSU], tRNAs, rRNAs, ORFs, other/hypothetical). Gene labels and orientation indicate the strand location; the inner gray track shows the GC content. (B) Circular mitochondrial genome (91,655 bp) with functional color coding (complex I NADH dehydrogenase, complex III ubiquinol–cytochrome c reductase, complex IV cytochrome c oxidase, ATP synthase, ribosomal proteins [SSU/LSU], other genes, tRNAs, rRNAs). Gene labels and orientation indicate strand location; the inner gray track shows GC content; gray arrows denote large repeat regions.

Supplementary Tables

Table S1. Distribution of syntenic and non-syntenic genes in *T. utriculariae*.

	**Genes in synteny blocks**	**Genes not in synteny blocks**	**Total**
***T. utriculariae*-specific genes**	1,792	716	2,508
Non-specific genes	19,651	1,060	20,711
**Total**	**21,443**	**1,776**	**23,219**

Table S2. Mitochondrial genome comparison among *Tetrahymena* species that were used in phylogenetic analysis.

Species	Genome Size (bp)	AT Content	Total Genes	Protein Coding Genes	tRNA Genes	rRNA Genes	Gene Density	Gene Proportion	Intergenic Proportion	Total Intergenic Length
T. utriculariae	51,725	78.53	58	44	7	6	1,121.31	87.67	12.63	6,534
T. thermophila	47,577	79.24	60	44	8	7	1,261.11	95.91	4.09	1,947
T. malaccensis	47,691	80.09	59	45	8	6	1,237.13	95.46	4.54	2,163
T. paravorax	47,496	81.53	60	44	10	6	1,263.26	94.62	5.65	2,684
T. pyriformis	47,296	78.67	58	45	8	6	1,226.32	95.75	4.25	2,011
T. rostrata	47,310	78.29	59	45	8	6	1,247.09	95.82	4.18	1,978

Table S3. Tajima's relative rate test results for mitochondrial DNA–encoded and nuclear-encoded mitochondrial proteins in *T. utriculariae* and close relatives, with corresponding IQ-TREE branch length measurements (related to Figure 2B and C).

Gene set	Comparison	Sequence A	Branch length (A)	Sequence B	Branch length (B)	Outgroup (C)	Branch length (C)	χ^2	df	*P* value	Interpretation
Mito DNA-encoded proteins	A vs B (C outgroup)	*T. utriculariae*	0.155	*T. malaccensis*	0.100	*T. thermophila*	0.098	95.55	1	<0.0001	Reject equal rates; *T. utriculariae* faster
Mito DNA-encoded proteins	A vs B (C outgroup)	*T. utriculariae*	0.155	*T. thermophila*	0.098	*T. malaccensis*	0.100	72.16	1	<0.0001	Reject equal rates; *T. utriculariae* faster
Nuclear-encoded mitochondrial proteins	A vs B (C outgroup)	*T. utriculariae*	0.059	*T. malaccensis*	0.032	*T. thermophila*	0.043	784.86	1	<0.0001	Reject equal rates; *T. utriculariae* faster
Nuclear-encoded mitochondrial proteins	A vs B (C outgroup)	*T. utriculariae*	0.059	*T. thermophila*	0.043	*T. malaccensis*	0.032	141.08	1	<0.0001	Reject equal rates; *T. utriculariae* faster

Table S4: List of mitochondrial reference genomes used to assemble the *M. tetrahymenae* mitochondrial genome.

Species	Strain/isolate
Micractinium sp	LBA 32
Chlorella sorokiniana	1230
Chlorella variabilis	NC64
Chlorella vulgaris	211-11P
Chlorella vulgaris	NJ-7

Table S5: List of chloroplast reference genomes used to assemble the *M. tetrahymenae* chloroplast genome.

Species	Strain/isolate
Micractinium conductrix	SAG-241.80
Chlorella sorokiniana	1230
Chlorella variabilis	NC64
Chlorella vulgaris	NC 001865.1
Chlorella vulgaris	NJ-7
