# Supplementary material for: Environment-dependent mutualism–parasitism transitions in the incipient symbiosis between Tetrahymena utriculariae and Micractinium tetrahymenae
Source: ISME J. 2025 Sep 6;19(1):wraf203. doi: 10.1093/ismejo/wraf203 (PMC12516953; doi:10.1093/ismejo/wraf203)
Supplement: ISMEJ-D-25-01009R2_Supplementary_text_wraf203 [file ismej-d-25-01009r2_supplementary_text_wraf203.pdf]

Environment-dependent mutualism-parasitism transitions in the incipient symbiosis between *Tetrahymena utriculariae* and *Micractinium tetrahymenae*

Kamal Md Mostafa[1#], Yu-Hsuan Cheng[1,†,#], Li-Wen Chu[1], Phuong-Thao Nguyen [1,2], Chien-Fu Jeff Liu[1], Chia-Wei Liao[1], Thomas Posch[3], Jun-Yi Leu[1,*]

[1] Institute of Molecular Biology, Academia Sinica, Taipei, 11529, Taiwan

[2] Department of Life Sciences, National Central University, Taoyuan, 320, Taiwan

[3] Limnological Station, Department of Plant and Microbial Biology, University of Zurich, Kilchberg, Switzerland

[#] These authors contributed equally.

[†] Present address: Morgridge Institute for Research, University of Wisconsin-Madison, Madison, WI, USA

[*] Corresponding author: Jun-Yi Leu, Institute of Molecular Biology, Academia Sinica, 128 Sec. 2, Academia Road, Taipei 115, Taiwan. Email: jleu@as.edu.tw

This file contains Supplementary Materials and Methods, legends, and captions for Supplementary Figures, Tables, and Data.

Supplementary Materials and Methods

Tetrahymena strains and culture conditions

To assess the growth of *T. utriculariae* under both oxic and hypoxic conditions, ~1,000 cells were seeded in 2 mL of lettuce medium in modified Dryl's solution [1], supplemented with *K. pneumoniae* (NBRC 100048 strain) for oxic cultures. These cultures were maintained at room temperature (RT) with a 12 h light/12 h dark cycle. For hypoxic cultures, the starting cell density was 1,000 cells/mL, and the cultures were maintained at 23°C under hypoxic conditions with a 12 h light/12 h dark cycle. Hypoxia was achieved by culturing the cells inside airtight glass vials filled with culture media containing *T. utriculariae*, thereby leaving no space for air. A parallel group of oxic and hypoxic cultures was maintained under constant darkness to study the effect of light and photosynthesis by the endosymbiotic algae *M. tetrahymenae*. These dark-treated cultures allowed us to evaluate how the algae contribute to the growth of *T. utriculariae* under varying light conditions. Multiple tubes containing identical starter cultures (2 mL of the same cell density and bacteria as food) were prepared to facilitate long-term growth analysis of hypoxic cultures. The hypoxic cultures were maintained for 70 days, with weekly cell counts. Three tubes were opened per condition for cell counting, and they were discarded after use to avoid reintroduction into the hypoxic environment.

For oxic cultures, 2 mL of fresh lettuce medium in modified Dryl's solution [1], containing *K. pneumoniae,* was added to each well on Day 3 to support growth. On Day 4, 1 mL of the same medium was added. On Day 5, 5 µL of *K. pneumoniae* grown in LB medium was added. To serve as controls for a sufficient food supply, parallel cultures of *T. thermophila* were grown under identical oxic conditions, as they grew much faster and consumed more bacteria, including those in the light/dark and constant-dark groups. This protocol facilitated a comparative evaluation of symbiotic and aposymbiotic *Tetrahymena* spp. under varying environmental and light conditions over an extended culture period.

Obtaining a culture of free-living *M. tetrahymenae*

M. tetrahymenae was isolated by plating endosymbiotic *T. utriculariae* on CA [2-4] agar plates. The culture plates were incubated at 23°C with a 12 h light/12 h dark cycle. Approximately two weeks later, a single colony was streaked onto CA + ampicillin (100 mg/mL) agar plates, and two weeks after that, an axenic single colony was inoculated into CA + ampicillin (100 mg/mL) liquid culture.

Genome sequencing, assembly, and annotation of *T. utriculariae*

T. utriculariae cells in early stationary phase were starved for 2 days, collected at 1,000 × g for 3 min, and washed twice with modified Dryl's solution [1]. Approximately 1 × 10^6 cells were lysed in equal volumes of 0.25 M TCMS buffer and 0.3% (w/v) NP-40. Genomic DNA was extracted with a QIAGEN Genomic-tip 20/G (Qiagen, Germany). DNA concentration was measured on a Qubit fluorometer (Invitrogen, USA), and quality was assessed on a Bioanalyzer 2100 (Agilent

Technologies, USA). Long-read data were generated on a PacBio Sequel System (Pacific Biosciences, USA) and a MinION System (Oxford Nanopore Technologies, UK). A 20 kb insert genomic library was prepared according to the manufacturer's protocol and sequenced on the Sequel System. An Oxford Nanopore ligation library (SQK-LSK109) was sequenced on a MinION. For short reads, paired-end libraries were sequenced on MiSeq and NextSeq System (Illumina, USA). All sequencing was performed at the Genomics Core Facility of the Institute of Molecular Biology, Academia Sinica. Illumina reads were quality-trimmed with Trimmomatic v0.38 [5] using the following options: ILLUMINACLIP=2:30:10 LEADING=3 TRAILING=3 SLIDINGWINDOW=4:15 MINLEN=36. PacBio subreads and Nanopore reads > 1 kb with mean quality > Q9 were assembled with Canu v2.0 [6] (corMaxEvidenceErate=0.15 genomeSize=100m). The initial assembly contained 2,089 contigs. Contigs predicted to be repeats or bubbles, circular contigs, and contigs < 10 kb were removed, yielding 518 contigs. Of these, 216 contigs with telomere repeats at ≥ 1 end were selected for further assembly. To collapse haplotypes, Redundans v0.14a [7] was used to generate a haploid assembly and exclude contigs representing two haplotypes of the same chromosome (alignment length > 90% of the shorter contig and identity > 90%). Redundans filtering produced 174 chromosome-level contigs and nine contigs with telomere repeats at one end. The 174 contigs were aligned to the *T. thermophila* reference assembly; contig 161 aligned to two chromosomes. Read mappings indicated that connecting reads spanning homologous regions likely derived from the micronuclear genome. Reads mapping to contig 161 and to the nine single-telomere contigs were reassembled with Canu v2.0 (correctedErrorRate=0.15, corMaxEvidenceErate=0.15, genomeSize=2m) [6], producing seven additional chromosome-level contigs. In total, the final assembly comprised 181 chromosome-level contigs. Polishing was performed with NextPolish v1.3.1 [8] using Nanopore reads and with Pilon v1.23 [8] using Illumina reads (three iterations each).

For gene prediction, AUGUSTUS v3.2.3 [9] was trained on the *T. thermophila* 2014 gene annotation obtained from the Tetrahymena Genome Database Wiki [10, 11], with parameter optimization via optimize_augustus.pl. Genome annotation was then carried out with BRAKER2 v2.1.4 [12] (options: AUGUSTUS_ab_initio --translation_table=6), combining RNA-seq hints and protein sequences from *T. thermophila*, *T. malaccensis*, *T. elliotti*, and *T. borealis* with the trained model.

Genome sequencing, assembly, and annotation of *M. tetrahymenae*

Genomic DNA was extracted using a modified CTAB-based method as described previously [13]. CTAB buffer contained 1% CTAB, 1.4 M NaCl, 100 mM Tris-HCl (pH 8), 1% Triton X-100, and 20 mM EDTA (pH 8). TE buffer contained 10 mM Tris-HCl (pH 8) and 1 mM EDTA (pH 8). A phenol/chloroform/isoamyl alcohol mixture (25:24:1) with 0.1% 8-hydroxyquinoline (PCIA) was used, together with RNase A (10 mg/mL), 100% ethanol, 70% ethanol, and 3 M sodium acetate (NaOAc, pH 5.2). Approximately 1 × 10^9 *M. tetrahymenae* cells were harvested and washed twice

with TE buffer by centrifugation at 10,000 × g for 30 s each time. Pelleted cells were resuspended in 750 µL CTAB extraction buffer and homogenized with an equal volume of 0.5 mm beads at 6 m/s for 80 s. The homogenate was centrifuged at maximum speed for 5 min, and the aqueous phase was transferred to a new tube. Proteinase K (160 µg/mL) was added, and the mixture was incubated at 56°C for 1 h. Next, 500 µL of 5 M NaCl was added and mixed thoroughly, followed by 400 µL of prewarmed (65°C) CTAB buffer. The solution was then incubated at 65°C for 10 min. The lysate was extracted three times with an equal volume of PCIA by mixing and centrifugation at maximum speed for 5 min, transferring the aqueous phase to a fresh tube each time. DNA was precipitated by adding 1 mL of 100% ethanol and 20 µL of NaOAc, followed by incubation at room temperature for 30 min. The sample was centrifuged at 16,000 × g for 5 min, and the supernatant was discarded. The pellet was washed with 100 µL of 70% ethanol and centrifuged for 1 min at 16,000 × g. It was then air-dried for 10 min and rehydrated in 400 µL of TE buffer containing 3 µL of RNase A, followed by incubation at 37°C for 1 h. Nucleic acids were reprecipitated with 1 mL 100% ethanol and 20 µL NaOAc at room temperature for 30 min, pelleted at 16,000 × g for 5 min, washed with 100 µL 70% ethanol (1 min at 16,000 × g), air-dried for 10 min, and rehydrated in 50 µL TE buffer. DNA integrity and quality were assessed on a Bioanalyzer 2100 (Agilent Technologies, USA).

Whole-genome sequencing used Illumina paired-end (PE) short reads and Oxford Nanopore Technologies (ONT) long reads. Illumina PE sequencing generated high-accuracy short reads, and ONT generated long reads for assembly. Read quality metrics (length and quality) were evaluated with NanoPlot [14]. *De novo* assembly was performed using Canu v2.0 [6] (options: minReadLength=5000, genomeSize=65m), which carried out read correction, trimming, and assembly via the best overlap graph algorithm. Assembly statistics (e.g., N50) were evaluated with QUAST [15]. The assembly was polished iteratively: three rounds with Pilon [8] using Illumina reads, followed by three rounds with NextPolish [16], integrating both long and short reads. Telomeric regions were identified with Tandem Repeat Finder (TRF) [17] in combination with custom scripts. Genome annotation used BRAKER2 [12] with RNA-seq evidence to improve gene prediction; GeneMark-ES [18] (embedded in BRAKER2) provided ab initio models. Genome completeness was assessed with BUSCO [19] using single-copy orthologs across genome, transcriptome, and proteome datasets.

Protein function prediction

Protein functions for *T. utriculariae* and *M. tetrahymenae* were predicted using multiple tools. InterProScan 5.40-77 [20] with the --goterms option assigned Gene Ontology (GO) terms to proteins. For *T. utriculariae*, functional annotation was expanded by inferring protein-protein interactions using DScript v0.2.2 [21] using the trained model topsy_turvy_v1.sav. An interaction-probability threshold of 0.92 was chosen so that the density of predicted positives (0.79%) matched that observed in *Saccharomyces cerevisiae* (0.78%) in BioGRID [22]. For genes lacking functional annotations, GO-

term enrichment among predicted interaction partners was tested gene-by-gene with Fisher's exact test and Bonferroni correction; significance was set at adjusted $P < 1 \times 10^{-10}$. This procedure yielded functional predictions for 4,769 previously unannotated genes.

For *M. tetrahymenae*, annotations were supplemented with MapMan Mercator4 v7.0 [23]. For genes still lacking functional assignments in either species, additional predictions were obtained with PANNZER2 [24], the OMA browser functional annotation tool [25], and STRING v12.0 [26]. In total, GO terms were assigned to 20,777 of 23,219 *T. utriculariae* genes and 13,630 of 14,229 *M. tetrahymenae* genes, substantially increasing functional coverage.

Synteny analysis

Synteny between *T. thermophila* and *T. utriculariae* was assessed using protein-level homology and collinearity. An all-versus-all homology search using protein sequences from both species was performed with DIAMOND v0.9.14 [27], using an E-value $\leq 1 \times 10^{-10}$ and retaining the top five hits per query. Collinear (syntenic) gene pairs were identified with MCScanX [28] via the DupGen_finder pipeline (GitHub: qiao-xin/DupGen_finder). Gene pairs assigned to whole-genome duplication (WGD) by MCScanX [28] were excluded to focus on non-WGD duplication modes. The remaining gene pairs were then classified by genomic location, facilitating the identification of conserved genomic regions and providing insights into genome evolution and structural conservation between *T. thermophila* and *T. utriculariae.*

Orthogroup identification

High-quality genome data for various *Tetrahymena* species (Supplementary figure S1A) were sourced from the *Tetrahymena* genome database [11] and the P10K database [29]. The freshwater obligate fish parasite *I. multifiliis*, a close relative of *Tetrahymena* spp. [30] was used as the outgroup. OrthoFinder (v2.5.5) [31], with a default inflation parameter of 1.5, was used to identify the orthologous genes among different ciliate species. Some of the orthogroups contained genes from only one species, and some genes that were not assigned to any orthogroups are defined as species-specific genes (Fig. 1C). Similar methods were used for *M. tetrahymenae* orthogroup prediction using the species mentioned in Figure 4A.

Molecular phylogeny and divergence time analysis

Single-copy orthogroups present in all species considered (for both the *T. utriculariae* and *M. tetrahymenae* datasets) were concatenated, aligned with MAFFT v7.525 [32], and trimmed using OrthoFinder default parameters. A maximum-likelihood tree was inferred with RAxML-NG [33] under the LG+G4 model from the concatenated alignment. A phylogenomic calibration from the ciliate fossil record places the split between *Ichthyophthirius* and *Tetrahymena* at ~450 Ma [34-36]. Divergence times were estimated in r8s [37] by calibrating the maximum-likelihood tree at that node.

Gene family expansion and contraction were evaluated with CAFE 5 [38] using a random birth-and-death model using OrthoVenn3 [39]. In addition, a focused OrthoFinder analysis was performed for the three closely related *Tetrahymena* species (*T. utriculariae*, *T. malaccensis*, and *T. thermophila*), with *T. elliotti* serving as the outgroup. Orthologous genes from this subset were used to assess changes in gene family size.

Amino acid identity analysis

Pairwise amino acid identity (AAI) was computed with CompareM v0.1.2 [40]. Runs used the proteins workflow with --proteins --keep_rbhs --cpus 32 and default thresholds (--evalue 1e-5, --per_identity 0.3, --per_aln_len 0.7). Reciprocal best hits (RBH) were retained for each species pair. To ensure comparability across comparisons, AAI values were calculated from a common set of 9,430 one-to-one orthologs per species pair (as defined in the orthogroup analysis).

Gene ontology enrichment

A customized org.db package for *T. utriculariae* and *M. tetrahymenae* was generated using the makeOrgPackage function from the Bioconductor [41] R [42] package AnnotationForge [43]. All GO term enrichment analyses were conducted using Clusterprofiler (v 4.10.1) [44], and GO term redundancy was reduced using REVIGO [45] with default parameters. The reduced set of GO terms was further selected based on enrichment scores.

Organellar genome assembly and annotation

The *T. utriculariae* mitochondrial genome was identified by searching Canu [6] contigs (excluding telomere-bearing contigs) with the *T. thermophila* mitochondrial sequence (BLAST). Contigs with significant matches were extracted, and reads mapping to those contigs were reassembled using Canu [6], followed by polishing with NextPolish and Pilon as described for the nuclear assembly. Annotation used AUGUSTUS [9] with hints generated by Exonerate [46] (-m protein2genome --maxintron 500 --geneticcode 4) from *T. thermophila* mitochondrial protein sequences. The training set was derived from annotations of the *T. thermophila* mitochondrial genes. The resulting FASTA and CDS outputs were further annotated and curated with GeSeq [47], MFannot [48], and Infernal (v1.1.5) [49]; the same tools were used to compare other *Tetrahymena* mitogenomes (Table 2). Nuclear-encoded mitochondrial proteins were identified by BLASTP using *T. utriculariae* proteins against the *T. thermophila* nuclear-encoded mitochondrial protein identified in a previous report [50] (*E* value < 1 × 10^-5). Orthologous groups for the identified proteins (n = 513) were then used to infer a species tree with STAG (Species Tree from All Genes) [51].

For *M. tetrahymenae*, the mitochondrial and chloroplast genomes were assembled using the same read-selection and reassembly approach, guided by reference organellar genomes (Supplementary Tables S4, S5). Annotation of both organelles used GeSeq [47].

Phylogenetic analysis of mitochondrial and nuclear-encoded mitochondrial proteins

Orthologous groups corresponding to mitochondrial-encoded genes and nuclear-encoded mitochondrial proteins were identified using OrthoFinder v2.5.4 [31] with the -M msa -T iqtree options, which align each orthogroup with MAFFT v7 [32] and infer gene trees with IQ-TREE. For concatenated phylogenies, protein alignments for each set were concatenated and analyzed with IQ-TREE v3.0.1 [52] under the LG+F+G4 substitution model [53]. Node support was assessed with Shimodaira-Hasegawa approximate likelihood ratio tests [54] and ultrafast bootstrap [55], each with 1,000 replicates. Branch lengths are reported as substitutions per site.

Tajima's relative rate test was performed to test for statistically significant differences in evolutionary rates between *T. utriculariae* and its closest relatives [56] on the concatenated protein alignments using MEGA11 [57]. All positions containing gaps and missing data were eliminated using the complete deletion option. The tests compared *T. utriculariae* against *T. malaccensis* and *T. thermophila* in reciprocal outgroup designs, with significance assessed at $\alpha = 0.05$.

Transmission electron microscopy (TEM) imaging

TEM sample preparation was conducted according to a microwave-assisted fixation protocol to examine the ultrastructure of mitochondria and interactions between perialgal vacuoles and symbiotic *T. utriculariae*. Cells were fixed in 2.5% glutaraldehyde and 4% paraformaldehyde in 100 mM sodium cacodylate buffer (pH 7.2) using eight cycles of microwave irradiation (500 W, 1 min) under vacuum, followed by room temperature incubation and overnight storage at 4°C. After buffer rinses, the samples underwent post-fixation with 1% osmium tetroxide using a similar microwave cycling protocol (450 W). Samples were then water-rinsed and stained *en bloc* with 2% uranyl acetate under microwave irradiation. Dehydration proceeded through an ethanol gradient (10-100%) with brief microwave treatments at each concentration. Infiltration was performed using Spurr's resin, starting at a 5% concentration and gradually increasing to 100% with vacuuming and regular agitation, followed by polymerization at 70°C for 24 hours. Ultrathin sections (60-90 nm) were collected on copper grids, stained with lead citrate, and imaged using a transmission electron microscope at 100 kV. Images were analyzed for mitochondrial morphology, perialgal vacuole associations, and ultrastructural differences between symbiotic and aposymbiotic cells.

Etomoxir treatment assay

To investigate the role of mitochondrial fatty acid oxidation (FAO) in *T. utriculariae*, pharmacological inhibition of FAO was performed using etomoxir (ETO), an irreversible inhibitor of carnitine palmitoyltransferase 1 [58] (CAS: 124083-20-1, MedChemExpress). *T. utriculariae* cells were cultured in 1 mL microtiter plates with an initial concentration of 10,000 cells/mL in 2.5% (v/v) lettuce medium supplemented with modified Dryl's solution, and K. pneumoniae (NBRC 100048)

was used as a food source. Cell cultures were at 23°C under a 12 h light/12 h dark cycle without agitation. Experimental groups received final concentrations of 0 μM (control with equivalent ddH_2O volume), 25 μM, or 50 μM ETO. Cell growth was monitored every 3 days by counting live cells under a microscope, with at least three biological replicates per condition to ensure reproducibility.

For endosymbiont number quantification, a slide-pressing method was used, in which appropriately diluted cells were placed on glass slides and gently pressed under a coverslip to lyse the host cells, allowing for clear visualization and accurate counting of the released *M. tetrahymenae* endosymbionts. Fluorescence microscopy (Axio Observer, Carl Zeiss) was used to determine chlorophyll autofluorescence, analyzing at least 50 host cells per condition for statistically robust quantification.

Transcriptome sequencing and analysis*: Tetrahymena utriculariae* RNA isolation

RNA was extracted from both symbiotic and aposymbiotic cells in the stationary phase using a combination of TRI reagent (Sigma, Merck, USA) and a Qiagen RNeasy mini kit (Qiagen, Germany). The cells were pelleted by centrifuging at 1000 × g for 3 minutes, after which the pellet was washed twice with modified Dryl's solution [1]. The cells were then resuspended in 200 μL nuclease-free water. To lyse the cells, 1 mL of TRI reagent was added to the suspended cells, and the mixture was gently pipetted to ensure proper homogenization. After incubating for a further 5 minutes at RT, 50 mM of potassium acetate was added to the mixture and incubated again for 5 minutes at room temperature. The algal endosymbionts and debris were separated from the rest of the mixture by centrifuging at 12000 × g for 10 minutes at 4°C. The resulting supernatant was transferred to a clean 2 mL tube, and then 300 μL of chloroform was added. The mixture was mixed vigorously for 3 minutes and then centrifuged at 21000 × g for at least 20 minutes at 4°C to separate the organic and aqueous phases. The upper aqueous phase was mixed with an equal volume of 100% ice-cold ethanol. The mixture was remixed by pipetting and transferred to Qiagen RNeasy columns. RNA extraction was performed according to the column manufacturer's instructions. RNA yield and purity were measured using a Qubit Fluorometer (Invitrogen, USA), and RNA integrity and quality were checked using a Bioanalyzer 2100 system (Agilent Technologies, USA).

Transcriptome sequencing and analysis: *Micractinium tetrahymenae* RNA isolation

Endosymbiotic *M. tetrahymena* were isolated from symbiotic, early stationary-phase *T. utriculariae* cells using a sonicator. The cells were washed five times with nuclease-free water. Free-living *M. tetrahymenae* at log phase were used as a control. Total mRNA was extracted using the same method as described above for *T. utriculariae*, with an additional step to break algal cells by adding 0.5 mL of 0.5 mm glass beads. Cells were beaten after adding 1 mL TRI reagent using an MP-Bio Fastprep-24 G bead beater at 6 ms^{-1} for 80 seconds, followed by 12000 × g centrifugation for 10 minutes at 4°C. The supernatant was transferred to a new tube and mixed vigorously for 3 minutes with 300 μL of

chloroform. The subsequent steps were the same as those described above to extract *T. utriculariae* mRNA.

Transcriptome sequencing and analysis: Library preparation and mRNA sequencing

Strand-specific RNA libraries were prepared with the SureSelect Strand-Specific RNA Library Preparation Kit for Illumina (Agilent Technologies) following the manufacturer's instructions. Libraries were sequenced on a NovaSeq 6000 System (Illumina) at Welgene Biotech Co., Ltd. (Taiwan), generating 150 bp paired-end reads for three biological replicates per condition (symbiotic and aposymbiotic). Quality control was performed using FastQC v0.11.8 [59], and adapter/quality trimming was performed with fastp v0.24.0 **[60]**. Transcript abundance was quantified with kallisto v0.51.0 (default parameters) [61].

Transcriptome sequencing and analysis: Differential gene expression analysis

Differential expression analyses were carried out separately for the host *T. utriculariae* and its endosymbiont *M. tetrahymenae* using the limma framework in R [42]. For each organism, the raw values of transcripts per million reads (TPM) were first filtered to retain only genes with a TPM value of ≥ 0.005 in at least 70% of the samples. The filtered counts were then $\log_2$-transformed (with a small pseudocount added to avoid zeros). The sample metadata were assembled to define, for *T. utriculariae*, the interaction of cell type (symbiotic vs. aposymbiotic) and time-point, and for *M. tetrahymenae*, the endosymbiotic status (Endo vs. Exo).

Linear models were fitted to the $\log_2$-TPM matrix via lmFit, and empirical Bayes moderation (eBayes) was applied to stabilize per-gene variance estimates and calculate moderated t-statistics [62]. For each gene and contrast, raw *P* values were obtained from the moderated t-distribution (with gene-specific, augmented degrees of freedom) and then adjusted using the Benjamini-Hochberg procedure to control the false discovery rate [63]. Genes exhibiting $|\log_2 \text{fold-change}| \geq 1$ and an adjusted *P* value ≤ 0.01 were classified as significantly up- or downregulated under the specified conditions.

Transcriptome sequencing and analysis: K-means clustering and heatmap visualization of *T. utriculariae* DEGs

Differentially expressed genes ($|\log_2 \text{fold-change}| \geq 1$, adjusted $P \leq 0.01$) were clustered on their z-score-normalized expression profiles across the 12 samples (six symbiotic, six aposymbiotic, using iDEP [64]). Clustering was performed using the KMeans_rcpp function in ClusterR [65], specifying k = 4 clusters with k-means++ initialization, 30 random restarts (num_init = 30), and up to 300 iterations per run (max_iters = 300). The set.seed(123) function ensured reproducibility. The resulting cluster assignments were visualized with the ComplexHeatmap package [66, 67], ordering rows by each gene's peak absolute expression and splitting columns by symbiotic status (symbiotic vs. aposymbiotic), with an overlaid annotation of light regime (dark vs. light).

Measuring chlorophyll content

Equal numbers of endosymbiotic and free-living *M. tetrahymenae* cells were harvested and pelleted (3,000 × g, 5 min, 4°C), followed by extraction in 1 mL of 100% methanol, with cells either ground in liquid nitrogen or bead-beaten in the dark for 20 minutes at 4°C. After clarification (14,000 × g, 5 min, 4°C), absorbance was measured using a 1 cm path-length cuvette at 652 nm (A_{652}) and 665 nm (A_{665}). Chlorophyll concentrations were calculated using the equations: Chlorophyll a (µg/mL) = $16.29 \times A_{665} - 8.54 \times A_{652}$; Chlorophyll b (µg/mL) = $30.66 \times A_{652} - 13.58 \times A_{665}$, and total chlorophyll was determined as the sum of Chlorophyll a and Chlorophyll b [68, 69]. Measurements were conducted in triplicate, and two-tailed t-tests were used for statistical comparisons.

Computational validation and figure preparation

Data analysis scripts and computational workflows were validated and organized using artificial intelligence platforms, specifically Claude (Anthropic) and ChatGPT (OpenAI), to enhance code quality, debugging, and reproducibility of analyses. These AI tools were used solely for script validation and organization; all data processing, statistical analyses, and scientific interpretations were performed and verified by the authors.

Figure preparation involved the initial generation using R and Python, as specified in the respective analysis sections, followed by panel organization, font standardization, and formatting adjustments using Affinity Designer (Serif Ltd.) to ensure consistency with journal requirements. All final figures, analyses, and conclusions remain the full responsibility of the authors.

Supplementary references

1. Dryl S. Antigenic transformation in *Paramecium aurelia* after homologous antiserum treatment during autogamy and conjugation. *J Protozool*. 1959;6:s96
2. Provasoli L, Pintner IJ. Artificial media for freshwater algae: problems and suggestions. In: Tryon CA Jr, Hartman RT, editors. *The Pymatuning Symposia in Ecology: The Ecology of Algae*. Special Publication No. 2. Pittsburgh, PA: Pymatuning Laboratory of Field Biology, University of Pittsburgh; 1960. pp 84-96.
3. Ichimura T. The *Closterium calosporum* complex from the Ryukyu Islands. Variation and taxonomical problems. *Mem Natl Sci Mus (Tokyo). 1974;7:89-102*
4. Provasoli L. Media and prospects for the cultivation of marine algae. *Cultures and Collections of Algae Proceedings of the US-Japan Conference, Hakone, September 1966*. 1968:63-75
5. Bolger AM, Lohse M, Usadel B. Trimmomatic: a flexible trimmer for Illumina sequence data. *Bioinformatics*. 2014;30:2114-2120. https://doi.org/10.1093/bioinformatics/btu170

6. Koren S, Walenz BP, Berlin K *et al.* Canu: scalable and accurate long-read assembly via adaptive k-mer weighting and repeat separation. *Genome Res*. 2017;27:722-736. https://doi.org/10.1101/gr.215087.116
7. Pryszcz LP, Gabaldón T. Redundans: an assembly pipeline for highly heterozygous genomes. *Nucleic Acids Res.* 2016;44:e113. https://doi.org/10.1093/nar/gkw294
8. Walker BJ, Abeel T, Shea T *et al.* Pilon: an integrated tool for comprehensive microbial variant detection and genome assembly improvement. *PLoS One*. 2014;9:e112963. https://doi.org/10.1371/journal.pone.0112963
9. Stanke M, Keller O, Gunduz I *et al.* AUGUSTUS: ab initio prediction of alternative transcripts. *Nucleic Acids Res*. 2006;34:W435-439. https://doi.org/10.1093/nar/gkl200
10. Hamilton EP, Kapusta A, Huvos PE *et al*. Structure of the germline genome of *Tetrahymena thermophila* and relationship to the massively rearranged somatic genome. *eLife.* 2016;5:e19090. https://doi.org/10.7554/eLife.19090
11. Stover NA, Krieger CJ, Binkley G *et al*. Tetrahymena Genome Database (TGD): a new genomic resource for *Tetrahymena thermophila* research. *Nucleic Acids Res.* 2006;34:D500-D503. https://doi.org/10.1093/nar/gkj054
12. Bruna T, Hoff KJ, Lomsadze A *et al.* BRAKER2: automatic eukaryotic genome annotation with GeneMark-EP+ and AUGUSTUS supported by a protein database. *NAR Genom Bioinform*. 2021;3:lqaa108. https://doi.org/10.1093/nargab/lqaa108
13. Jagielski T, Gawor J, Bakula Z *et al.* An optimized method for high quality DNA extraction from microalga Prototheca wickerhamii for genome sequencing. *Plant Methods*. 2017;13:77. https://doi.org/10.1186/s13007-017-0228-9
14. De Coster W, D'Hert S, Schultz DT *et al.* NanoPack: visualizing and processing long-read sequencing data. *Bioinformatics*. 2018;34:2666-69. https://doi.org/10.1093/bioinformatics/bty149
15. Gurevich A, Saveliev V, Vyahhi N *et al.* QUAST: quality assessment tool for genome assemblies. *Bioinformatics*. 2013;29:1072-1075. https://doi.org/10.1093/bioinformatics/btt086
16. Hu J, Fan J, Sun Z *et al.* NextPolish: a fast and efficient genome polishing tool for long-read assembly. *Bioinformatics*. 2020;36:2253-55. https://doi.org/10.1093/bioinformatics/btz891
17. Benson G. Tandem repeats finder: a program to analyze DNA sequences. *Nucleic Acids Res*. 1999;27:573-580. https://doi.org/10.1093/nar/27.2.573
18. Borodovsky M, Lomsadze A. Eukaryotic gene prediction using GeneMark.hmm-E and GeneMark-ES. *Curr Protoc Bioinformatics*. 2011;Chapter 4:4.6.1-4.6.10. https://doi.org/10.1002/0471250953.bi0406s35

19. Simao FA, Waterhouse RM, Ioannidis P *et al.* BUSCO: assessing genome assembly and annotation completeness with single-copy orthologs. *Bioinformatics*. 2015;31:3210-3212. https://doi.org/10.1093/bioinformatics/btv351

20. Jones P, Binns D, Chang HY *et al.* InterProScan 5: genome-scale protein function classification. *Bioinformatics*. 2014;30:1236-12340. https://doi.org/10.1093/bioinformatics/btu031

21. Sledzieski S, Singh R, Cowen L *et al*. D-SCRIPT translates genome to phenome with sequence-based, structure-aware, genome-scale predictions of protein-protein interactions. *Cell Syst.* 2021;12:969-982.e6. https://doi.org/10.1016/j.cels.2021.08.010

22. Oughtred R, Rust J, Chang C *et al.* The BioGRID database: A comprehensive biomedical resource of curated protein, genetic, and chemical interactions. *Protein Sci*. 2021;30:187-200. https://doi.org/10.1002/pro.3978

23. Schwacke R, Ponce-Soto GY, Krause K *et al.* MapMan4: A Refined Protein Classification and Annotation Framework Applicable to Multi-Omics Data Analysis. *Mol Plant*. 2019;12:879-92. https://doi.org/10.1016/j.molp.2019.01.003

24. Toronen P, Medlar A, Holm L. PANNZER2: a rapid functional annotation web server. *Nucleic Acids Res*. 2018;46:W84-W88. https://doi.org/10.1093/nar/gky350

25. Altenhoff AM, Train CM, Gilbert KJ *et al*. OMA orthology in 2021: website overhaul, conserved isoforms, ancestral gene order and more. *Nucleic Acids Res.* 2021;49:D373-D379. https://doi.org/10.1093/nar/gkaa1007

26. Szklarczyk D, Kirsch R, Koutrouli M *et al*. The STRING database in 2023: protein-protein association networks and functional enrichment analyses for any sequenced genome of interest. *Nucleic Acids Res.* 2023;51:D638-D646. https://doi.org/10.1093/nar/gkac1000

27. Buchfink B, Reuter K, Drost HG. Sensitive protein alignments at tree-of-life scale using DIAMOND. *Nat Methods*. 2021;18:366-368. https://doi.org/10.1038/s41592-021-01101-x

28. Wang Y, Tang H, Debarry JD *et al.* MCScanX: a toolkit for detection and evolutionary analysis of gene synteny and collinearity. *Nucleic Acids Res*. 2012;40:e49. https://doi.org/10.1093/nar/gkr1293

29. Gao X, Chen K, Xiong J *et al*. The P10K database: a data portal for the protist 10 000 genomes project. *Nucleic Acids Res.* 2024;52:D747-D755. https://doi.org/10.1093/nar/gkad992

30. Coyne RS, Hannick L, Shanmugam D *et al.* Comparative genomics of the pathogenic ciliate *Ichthyophthirius multifiliis,* its free-living relatives and a host species provide insights into adoption of a parasitic lifestyle and prospects for disease control. *Genome Biol*. 2011;12:R100. https://doi.org/10.1186/gb-2011-12-10-r100

31. Emms DM, Kelly S. OrthoFinder: phylogenetic orthology inference for comparative genomics. *Genome Biol*. 2019;20:238. https://doi.org/10.1186/s13059-019-1832-y

32. Katoh K, Misawa K, Kuma K *et al*. MAFFT: a novel method for rapid multiple sequence alignment based on fast Fourier transform. *Nucleic Acids Res.* 2002;30:3059-3066. https://doi.org/10.1093/nar/gkf436
33. Kozlov AM, Darriba D, Flouri T *et al*. RAxML-NG: a fast, scalable and user-friendly tool for maximum likelihood phylogenetic inference. *Bioinformatics.* 2019;35:4453-4455. https://doi.org/10.1093/bioinformatics/btz305
34. Jiang CQ, Wang GY, Xiong J *et al.* Insights into the origin and evolution of Peritrichia (Oligohymenophorea, Ciliophora) based on analyses of morphology and phylogenomics. *Mol Phylogenet Evol*. 2019;132:25-35. https://doi.org/10.1016/j.ympev.2018.11.018
35. Xiong J, Yang W, Chen K *et al.* Hidden genomic evolution in a morphospecies-The landscape of rapidly evolving genes in Tetrahymena. *PLoS Biol*. 2019;17:e3000294. https://doi.org/10.1371/journal.pbio.3000294
36. Weitschat W, Guhl W. Erster Nachweis fossiler Ciliaten. *PalZ.* 1994;68:17-31. https://doi.org/10.1007/BF02989430
37. Sanderson MJ. r8s: inferring absolute rates of molecular evolution and divergence times in the absence of a molecular clock. *Bioinformatics*. 2003;19:301-302. https://doi.org/10.1093/bioinformatics/19.2.301
38. Mendes FK, Vanderpool D, Fulton B *et al*. CAFE 5 models variation in evolutionary rates among gene families. *Bioinformatics.* 2021;36:5516-5518. https://doi.org/10.1093/bioinformatics/btaa1022
39. Sun J, Lu F, Luo Y *et al*. OrthoVenn3: an integrated platform for exploring and visualizing orthologous data across genomes. *Nucleic Acids Res.* 2023;51:W397-W403. https://doi.org/10.1093/nar/gkad313
40. Parks DH. CompareM [Computer software]. GitHub. Version 0.1.2 https://github.com/dparks1134/CompareM. Accessed 20 Oct 2024.
41. Gentleman RC, Carey VJ, Bates DM *et al.* Bioconductor: open software development for computational biology and bioinformatics. *Genome Biol*. 2004;5:R80. https://doi.org/10.1186/gb-2004-5-10-r80
42. Ritchie ME, Phipson B, Wu D *et al.* limma powers differential expression analyses for RNA-sequencing and microarray studies. *Nucleic Acids Res*. 2015;43:e47. https://doi.org/10.1093/nar/gkv007
43. Carlson M, Pagès H. AnnotationForge: Tools for building SQLite-based annotation data packages [Computer software]. Bioconductor. Version 1.50.0. https://bioconductor.org/packages/AnnotationForge/. Accessed 12 Dec 2024. https://doi.org/10.18129/B9.bioc.AnnotationForge
44. Wu T, Hu E, Xu S *et al*. clusterProfiler 4.0: a universal enrichment tool for interpreting omics data. *The Innovation.* 2021;2:100141. https://doi.org/10.1016/j.xinn.2021.100141

45. Supek F, Bosnjak M, Skunca N *et al.* REVIGO summarizes and visualizes long lists of gene ontology terms. *PLoS One*. 2011;6:e21800. https://doi.org/10.1371/journal.pone.0021800
46. Slater GSC, Birney E. Automated generation of heuristics for biological sequence comparison. *BMC Bioinformatics*. 2005;6:31. https://doi.org/10.1186/1471-2105-6-31
47. Tillich M, Lehwark P, Pellizzer T *et al.* GeSeq—versatile and accurate annotation of organelle genomes. *Nucleic Acids Res.* 2017;45:W6-W11. https://doi.org/10.1093/nar/gkx391
48. Lang BF, Beck N, Prince S *et al.* Mitochondrial genome annotation with MFannot: a critical analysis of gene identification and gene model prediction. *Front Plant Sci*. 2023;14:1222186. https://doi.org/10.3389/fpls.2023.1222186
49. Nawrocki EP, Eddy SR. Infernal 1.1: 100-fold faster RNA homology searches. *Bioinformatics*. 2013;29:2933-2935. https://doi.org/10.1093/bioinformatics/btt509
50. Smith DG, Gawryluk RM, Spencer DF *et al.* Exploring the mitochondrial proteome of the ciliate protozoon Tetrahymena thermophila: direct analysis by tandem mass spectrometry. *J Mol Biol*. 2007;374:837-863. https://doi.org/10.1016/j.jmb.2007.09.051
51. Emms DM, Kelly S. STAG: Species Tree Inference from All Genes. *bioRxiv*. 2018:267914. https://doi.org/10.1101/267914
52. Wong T, Ly-Trong N, Ren H *et al.* IQ-TREE 3: phylogenomic inference software using complex evolutionary models. *EcoEvoRxiv.* 2025. https://doi.org/10.32942/x2p62n
53. Le SQ, Gascuel O. An improved general amino acid replacement matrix. *Mol Biol Evol*. 2008;25:1307-1320. https://doi.org/10.1093/molbev/msn067
54. Guindon S, Dufayard JF, Lefort V *et al.* New algorithms and methods to estimate maximum-likelihood phylogenies: assessing the performance of PhyML 3.0. *Syst Biol*. 2010;59:307-321. https://doi.org/10.1093/sysbio/syq010
55. Hoang DT, Chernomor O, von Haeseler A *et al.* UFBoot2: Improving the Ultrafast Bootstrap Approximation. *Mol Biol Evol*. 2018;35:518-522. https://doi.org/10.1093/molbev/msx281
56. Tajima F. Simple methods for testing the molecular evolutionary clock hypothesis. *Genetics*. 1993;135:599-607. https://doi.org/10.1093/genetics/135.2.599
57. Tamura K, Stecher G, Kumar S. MEGA11: Molecular Evolutionary Genetics Analysis Version 11. *Mol Biol Evol*. 2021;38:3022-3027. https://doi.org/10.1093/molbev/msab120
58. Lopaschuk GD, Wall SR, Olley PM *et al.* Etomoxir, a carnitine palmitoyltransferase I inhibitor, protects hearts from fatty acid-induced ischemic injury independent of changes in long chain acylcarnitine. *Circ Res*. 1988;63:1036-1043. https://doi.org/10.1161/01.res.63.6.1036
59. Andrews S. FastQC: A quality control tool for high throughput sequence data [Computer software]. Babraham Bioinformatics. https://www.bioinformatics.babraham.ac.uk/projects/fastqc/. 2010.

60. Chen S, Zhou Y, Chen Y *et al*. fastp: an ultra-fast all-in-one FASTQ preprocessor. *Bioinformatics.* 2018;34:i884-i890. https://doi.org/10.1093/bioinformatics/bty560
61. Bray NL, Pimentel H, Melsted P *et al.* Near-optimal probabilistic RNA-seq quantification. *Nat Biotechnol*. 2016;34:525-527. https://doi.org/10.1038/nbt.3519
62. Smyth GK. Linear models and empirical bayes methods for assessing differential expression in microarray experiments. *Stat Appl Genet Mol Biol*. 2004;3:Article3. https://doi.org/10.2202/1544-6115.1027
63. Benjamini Y, Hochberg Y. Controlling the false discovery rate - a practical and powerful approach to multiple testing. *J R Stat Soc Series B Stat Methodol*. 1995;57:289-300. https://doi.org/10.1111/j.2517-6161.1995.tb02031.x
64. Ge SX, Son EW, Yao R. iDEP: an integrated web application for differential expression and pathway analysis of RNA-Seq data. *BMC Bioinformatics*. 2018;19:534. https://doi.org/10.1186/s12859-018-2486-6
65. Mouselimis L. ClusterR: Gaussian Mixture Models, K-Means, Mini-Batch-Kmeans, K-Medoids and Affinity Propagation Clustering [Computer software]. CRAN. Version 1.3.3. https://CRAN.R-project.org/package=ClusterR. 2024. https://doi.org/10.32614/CRAN.package.ClusterR
66. Gu Z. Complex heatmap visualization. *iMeta.* 2022;1:e43. https://doi.org/10.1002/imt2.43
67. Gu Z, Eils R, Schlesner M. Complex heatmaps reveal patterns and correlations in multidimensional genomic data. *Bioinformatics*. 2016;32:2847-2849. https://doi.org/10.1093/bioinformatics/btw313
68. Porra RJ, Thompson WA, Kriedemann PE. Determination of accurate extinction coefficients and simultaneous equations for assaying chlorophylls a and b extracted with four different solvents: verification of the concentration of chlorophyll standards by atomic absorption spectroscopy. *Biochim Biophys Acta Bioenerg.* 1989;975:384-394. https://doi.org/10.1016/S0005-2728(89)80347-0
69. Ritchie RJ. Consistent sets of spectrophotometric chlorophyll equations for acetone, methanol and ethanol solvents. *Photosynth Res*. 2006;89:27-41. https://doi.org/10.1007/s11120-006-9065-9

Supplementary Figure captions and legends

Supplementary Figure S1: Phylogenetic relationships and evolutionary timeline of *Tetrahymena* species.

(A) Maximum-likelihood phylogeny for 12 *Tetrahymena* species with *Ichthyophthirius multifiliis* as outgroup, inferred from a concatenated alignment of single-copy orthologs identified with OrthoFinder. Branch lengths indicate genetic distance (scale bar: 0.10 substitutions per site). *T. utriculariae* forms a clade with *T. malaccensis* and *T. thermophila*. (B) Time-calibrated tree showing divergence times (Ma) for major *Tetrahymena* lineages. The genus originated ~447 million years ago (Ma), with diversification occurring near 380 Ma (Paravorax divergence) and 190 Ma (Borealis-Australis split). Within Borealis, *T. utriculariae* diverged from *T. thermophila* ~23 Ma and from *T. malaccensis* ~19 Ma.

Supplementary Figure S2: GO enrichment analysis of *T. utriculariae* gene categories: *T. utriculariae*-specific, expanded, and contracted genes.

Enriched GO terms for *T. utriculariae*-specific genes, expanded gene families, and contracted gene families. *T. utriculariae*-specific and expanded sets are enriched for ion transport/homeostasis, signal transduction, protein modification, and stress responses; contracted families are enriched for developmental processes and cell adhesion. Circle size denotes the enrichment score, and color encodes the adjusted *P* value.

Supplementary Figure S3: Comparative genomic architecture reveals chromosomal evolution between *T. utriculariae* and *T. thermophila*.

(A) Telomeric species-specific genes: chromosome 120 comparison shows a conserved central syntenic region (green/blue blocks) and terminal *T. utriculariae*-specific genes (purple). (B) Fusion region: material from two *T. thermophila* chromosomes (Tt Chr 31 and Tt Chr 29) corresponds to a single *T. utriculariae* chromosome (Tu Chr 31); syntenic blocks in green/blue; non-syntenic species-specific genes in purple. (C) Expanded region: *T. utriculariae* chromosome 64 is longer than its *T. thermophila* counterpart; paralogous, duplication-derived genes are shown in dark blue.

Supplementary Figure S4: Distinct mitochondrial morphologies in aposymbiotic versus symbiotic *T. utriculariae* cells. Continuation from Figure 2D.

(A) Aposymbiotic cell: mitochondria (pseudocolored blue) display rounded to oval morphologies throughout the cytoplasm. Main image scale bar: 2 µm; inset scale bar: 0.5 µm. (B) Symbiotic cell containing *M. tetrahymenae* (green pseudocolor): mitochondria (blue) are elongated and closely associated with the perialgal vacuole membrane (PVM; white arrow). Main image scale bar: 2 µm; inset scale bar: 0.5 µm. (C) Gallery of symbiotic cells maintained under low oxygen, showing

consistent mitochondria-perialgal vacuole associations; mitochondria in blue pseudocolor and endosymbionts in green. Scale bars (white line inside black box): 0.5 µm.

Supplementary Figure S5: Organellar genome architecture of the endosymbiotic alga *M. tetrahymenae*.

(A) Circular plastid genome (133,089 bp) with genes color-coded by function (photosystems I/II, cytochrome b/f, ATP synthase, assembly/stability factors, RNA polymerase, ribosomal proteins [SSU/LSU], tRNAs, rRNAs, ORFs, other/hypothetical). Gene labels and orientation indicate the strand location; the inner gray track shows the GC content. (B) Circular mitochondrial genome (91,655 bp) with functional color coding (complex I NADH dehydrogenase, complex III ubiquinol-cytochrome c reductase, complex IV cytochrome c oxidase, ATP synthase, ribosomal proteins [SSU/LSU], other genes, tRNAs, rRNAs). Gene labels and orientation indicate strand location; the inner gray track shows GC content; gray arrows denote large repeat regions.

Supplementary Tables captions

Table S1. Distribution of syntenic and non-syntenic genes in *T. utriculariae*.

Table S2. Mitochondrial genome comparison among *Tetrahymena* species that were used in phylogenetic analysis.

Table S3. Tajima's relative rate test results for mitochondrial DNA-encoded and nuclear-encoded mitochondrial proteins in *T. utriculariae* and close relatives, with corresponding IQ-TREE branch length measurements (related to Figure 2B and C).

Table S4: List of mitochondrial reference genomes used to assemble the *M. tetrahymenae* mitochondrial genome.

Table S5: List of chloroplast reference genomes used to assemble the *M. tetrahymenae* chloroplast genome.

Supplementary Data

Supplementary Data S1: Orthogroup Analysis of *Tetrahymena* Species

S1A. *T. utriculariae* gene info

Detailed annotation of *T. utriculariae* genes, including gene IDs, descriptions, symbols, UniProt IDs, and mini chromosome IDs.

S1B. Shared by all three *Tetrahymena* species

Orthologous gene clusters, identified through OrthoFinder, are shared among *Tetrahymena utriculariae*, *T. malaccensis*, and *T. thermophila*.

S1C. Specific to *T. utriculariae*

Genes exclusively identified in the *T. utriculariae* genome, annotated with gene IDs and descriptions.

Supplementary Data S2: Genome-wide Gene Ontology (GO) and Gene Family Analysis of *Tetrahymena utriculariae*

S2A. *T. utriculariae*-specific GO

Gene Ontology enrichment analysis of *T. utriculariae*-specific genes.

S2B. *T. utriculariae* expanded gene families

Expanded gene families identified in *T. utriculariae* via CAFE5 analysis.

S2C. *T. utriculariae* contracted gene families

Contracted gene families identified in *T. utriculariae* via CAFE5 analysis.

S2D. Expanded GO

Gene ontology enrichment analysis for expanded gene families in *T. utriculariae*.

S2E. Contracted GO

Gene ontology enrichment analysis for contracted gene families in *T. utriculariae*.

Supplementary Data S3: Transcriptomic Analysis of *Tetrahymena utriculariae*

Metadata

Metadata information describing RNA-seq samples, conditions, biological replicates, symbiotic states, and time-points.

S3A. TPM

Transcripts per million (TPM) data for *T. utriculariae* genes in symbiotic and aposymbiotic states across multiple diurnal time-points with biological replicates.

S3B. Differentially expressed genes

Differentially expressed genes (DEGs) between symbiotic and aposymbiotic conditions, including statistical parameters (fold-change, adjusted p-values).

S3C. K-means clustering

Expression profiles of DEGs were grouped by k-means clustering into different temporal and condition-specific clusters.

S3D. *T. utriculariae* gene ontology

Gene ontology enrichment analyses for each DEG cluster, detailing functional implications in symbiotic versus aposymbiotic states.

Supplementary Data S4: Transcriptomic Analysis of *Micractinium tetrahymenae*

***M. tetrahymenae* gene information**

Annotation details for *M. tetrahymenae* genes, including gene IDs, descriptions, standardized symbols, and UniProt IDs.

S4A. *M. tetrahymenae* TPM

Transcripts per million (TPM) data comparing endosymbiotic and free-living *M. tetrahymenae* conditions.

S4B. *M. tetrahymenae* differentially expressed genes

Differentially expressed genes (DEGs) between endosymbiotic and free-living conditions, with expression statistics and significance.

S4C. *M. tetrahymenae* gene ontology

Gene ontology enrichment analysis highlighting key metabolic and physiological shifts in endosymbiotic *M. tetrahymenae* compared to the free-living state.
